# Supplementary material for: eQTL mapping using allele-specific count data is computationally feasible, powerful, and provides individual-specific estimates of genetic effects
Source: PLoS Genet. 2022 Mar 14;18(3):e1010076. doi: 10.1371/journal.pgen.1010076 (PMC8947591; doi:10.1371/journal.pgen.1010076)
Supplement: S1 Text — Supplementary Materials for methods and results, including Figs A1-A36, and Tables A1-A18. (PDF) [file pgen.1010076.s007.pdf]

Supplementary Materials for "eQTL mapping using allele-specific gene expression"

Contents

A Data processing pipeline **3**
A.1 Geuvadis dataset 3
A.1.1 Genotype phasing and imputation 4
A.1.2 Processing RNA-seq 5
A.2 GTEx v7 dataset 7
A.3 GTEx v8 dataset 8
A.3.1 Genotype and covariates data 8
A.3.2 Processing RNA-seq on a cloud computing platform 9

B Supplementary Methods **11**
B.1 Probability distributions used by TReCASE and RASQUAL 11
B.1.1 TReCASE definition 11
B.1.2 RASQUAL definition 13
B.1.3 Definition of RASQUAL-like method: TReCASE-RL 16
B.1.4 The over-dispersion parameters of the three models 16
B.2 Simulation setup 17
B.2.1 Simulation for TReC 17
B.2.2 Simulation for ASReCs without within-sample over-dispersion 17
B.2.3 Add within-sample over-dispersion for ASReC data 18
B.2.4 To simulate RASQUAL style ASReC data 19
B.2.5 Simulating the data with genotyping errors 19

C Supplementary Results **20**
C.1 Estimation of permutation p-value estimation using geoP 20
C.1.1 Evaluation of geoP using Geuvadis dataset 20
C.1.2 Evaluation of geoP using GTEx v7 dataset 23

C.2 Use simulations to compare the power of TReCASE vs. MatrixEQTL. 25
C.3 Supplementary results for GTEx v8 data. 26
C.3.1 Results of gene-SNP pairs 26
C.3.2 Examples of the eQTLs missed by MatrixEQTL but captured by TReCASE 29
C.3.3 GWAS enrichment 32
C.4 Supplementary results for dynamic eQTLs. 40
C.4.1 Cell type proportion estimate from whole blood 40
C.4.2 Dynamic eQTLs related with CTCF and TP53 40
C.5 Evaluation of the binomial distribution assumption for ASReCs across multiple SNPs within one gene and one sample 42
C.6 Simulation Results to compare TReCASE vs. RASQUAL 43
C.6.1 Simulation results under RASQUAL assumption 43
C.6.2 Simulations results with within sample over-dispersion and additional between sample over-dispersion for ASReCs 45
C.6.3 Evaluating models for the data simulated under TReCASE assumption 45
C.6.4 Evaluation of the inflation of type I error by RASQUAL . . . 47
C.6.5 Compare TReCASE versus RASQUAL when there are genotyping errors 52
C.7 Compare MatrixEQTL, TReCASE, and RASQUAL using Geuvadis dataset 54
C.7.1 Computational time 54
C.7.2 Choose a permutation p-value cutoff to control FDR 57
C.7.3 Compare TReCASE vs. MatrixEQTL 58
C.7.4 Genomic locations of eQTLs 60
C.7.5 Compare the results of RASQUAL vs. TReCASE 60
C.7.6 eQTL mapping using permuted genotype data 69
C.8 Compare MatrixEQTL vs. TReCASE using Geuvadis and GTEx v7 data 73

A Data processing pipeline

Before going into the details of data processing steps for processing genotype and gene expression data from each dataset, we first illustrate a general pipeline (Fig A1). The steps of obtaining TReC per gene and per sample (the left part of the figure) and phased and imputed genotypes (the right side of the figure) are often standard steps in all the gene expression quantitative trait locus (eQTL) analyses. The middle section of the figure shows the steps of obtaining allele-specific reads at gene or SNP level, and it is an extra step to prepare data for eQTL mapping using allele-specific expression (ASE, or allele-specific read count, ASReC).

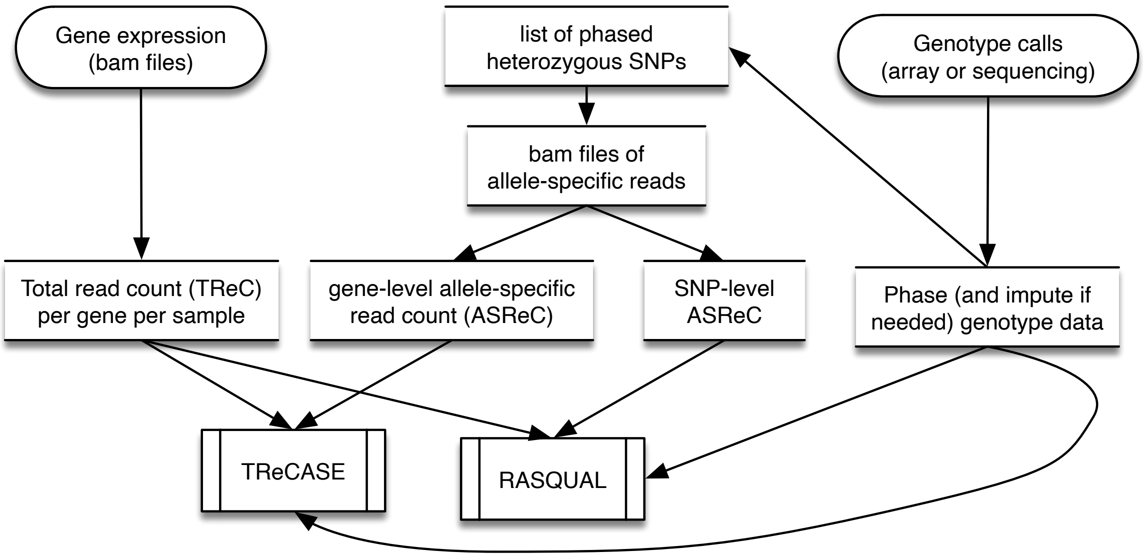

Fig. A1: Data processing pipeline

A.1 Geuvadis dataset

The 280 samples of this eQTL dataset are lymphoblastoid cell lines that are part of the samples used in 1000 Genomes Project (1KGP) [1]. The genotype data were obtained from SNP arrays and RNA-seq data were generated by the Geuvadis project [2].

A.1.1 Genotype phasing and imputation

SNP genotype data were obtained from the same Geuvadis project. Using SHAPEIT2 [3] and IMPUTE2 [4, 5], we phased and imputed genotypes in the following steps.

1. Convert unphased genotypes to PED/MAP format and ensure that genomic positions are converted to the genome reference that match the reference panel. This coordinate conversion can conveniently be done using liftover tool [6].

2. Run SHAPEIT2 in check mode to get a list of mismatching SNPs. We have observed that there are a notable portion of SNPs labeled as strand mismatch after this step. To fix this problem we flip the SNPs with Plink [7], repeat phasing in a check mode and compare resulting lists. If some of the SNPs present in first error list disappear in the second list they should be flipped and kept, and the rest of the mismatched SNPs can be supplied to SHAPEIT2 at the next step as an exclusion list.

3. Run SHAPEIT2 with exclusion list obtained in previous step.

4. Impute the pre-phased genotype data using IMPUTE2.

5. Using the imputed and phased data from the previous step, we obtain all heterozygous SNPs for each sample. Specifically, the main output file of imputation has 3 columns with genotype probabilities per SNP, which represent the probability of observing genotype $G = 0$, 1, or 2 respectively. We selected heterozygous locations (i.e., with high probability of $G = 1$) to output phased genotypes of these locations.

For both phasing and imputation, we used the 1000 Genome reference panel [4] (as of summer 2015) containing 2,504 samples with ∼82 million SNPs. Effective size of the population was set to the suggested value `--effective-size 20000` and random seed was set to 1234567. Imputation was done by splitting the genome into blocks of 5 Mb (no more than 7 Mb according to the instruction). We also used the same population size option as the one used in phasing step (`-Ne 20,000`), other options used inlcude `-align_by_maf_g` and `-seed 12345`.

The input data are genotypes of $\sim$2.2 million SNPs per sample for 2,123 samples of African or European descent. After phasing and imputation, we ended up with genotype data for $\sim$82 million SNPs per sample that include $\sim$2.2 million heterozygous SNPs per sample. Among all the imputed SNPs, around 6.5 million SNPs have MAF > 0.05 and were used as candidate eQTLs for eQTL mapping.

A.1.2 Processing RNA-seq

We downloaded raw RNA-seq data (in fastq format) of 462 samples that are part of the samples for the 1KGP [1]. These RNA-seq data were generated by the Geuvadis consortium [2], and it is available at `http://www.ebi.ac.uk/arrayexpress/experiments/E-GEUV-1/samples/`.

The RNA-samples were sequenced by the Illumina HiSeq2000 platform, with paired-end 75-bp reads. We mapped these RNA-seq reads to hg38 reference assembly using TopHat v2 [8], filtered with the following criteria: ≤ 3 mismatches, read gap length ≤ 3, read edit distance ≤ 3, and read realign edit distance equals to 0.

We filtered RNA-seq reads with average base quality ≥ 30, mapping quality ≥ 20, and keeping only those uniquely mapped reads, by the `prepareBAM` function from R package `asSeq`. In a typical sample, most of the RNA-seq reads pass these filters.

For each sample, given the list of heterozygous SNPs obtained from phased and imputed genotype data, we filter out allele-specific reads using R function `extractASReads` from `asSeq` package [9]. This step generates 3 bam files for each sample labeled with $hap1$, $hap2$ or $hapN$, which contain the RNA-seq reads that match haplotype 1, haplotype 2, or with conflicting information. Which haplotype is defined as haplotype 1 or haplotype 2 in each sample is arbitrary. The size of $hapN$ file should be much smaller than $hap1$ or $hap2$. Otherwise, there may be some systematic mismatch between RNA-seq bam files and the SNP list, e.g., they were generated using different human genome references. Apparent imbalance in $hap1$ and $hap2$ file sizes often suggest some problems in the data preparation steps.

Next we obtained the Total Read Count (TReC) and allele-specific read count (ASReC) per gene using R function `summarizeOverlaps` from `GenomicAlignments` package [10] using Gencode version 21 (GRCh38).We observed that for some samples, many genes have extreme proportions of reads attributed to one haplotype - this is likely due inconsistency between genotype data and RNA-seq data. We discarded such samples and used the remaining 280 samples for eQTL mapping. The total number of reads mapped to genes in these 280 samples vary from 16 to 82 million with fraction of allele-specific reads from 2.6% to 6.3% (Fig A2).

For RASQUAL we produced SNP level allele-specific counts using the same imputed SNPs and mapped bam files as input to `ASEReadCounter` from GATK package [11].

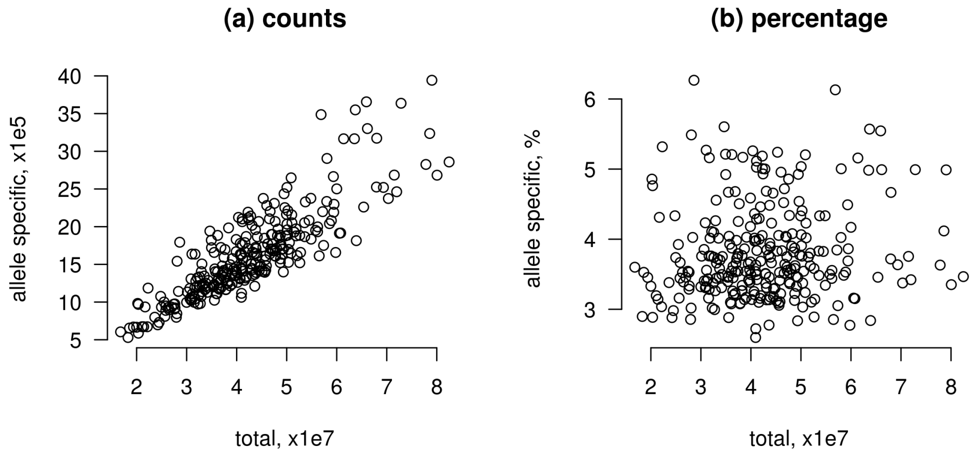

Fig. A2: Summary of total read counts (x-axis) versus total number of allele-specific reads (a) or the percentage of reads being allele-specific (b) across all genes per sample for the Geuvadis data. Each point indicates one of the 280 samples.

A.2 GTEx v7 dataset

All the eQTL results reported in our paper are based on GTEx v8 data. Though when we first started this project, the v8 data were not available and we use the whole blood data from v7 [12] to compare different computational methods. Here we briefly described how the v7 data were processed. We downloaded mapped RNA-seq data (in bam format) of 427 whole blood samples through NCBI Sequence Read Archive (SRA) after applying for data access through dbGaP (access id phs000424.v7.p2). The RNA-samples were sequenced by the Illumina HiSeq2000 platform with paired-end 76-bp reads and were mapped to human genome references hg19. We filtered RNA-seq reads with average base quality ≥ 20, mapping quality ≥ 20, and keeping only those uniquely mapped reads, by the `prepareBAM` function from R package `asSeq`.

We downloaded genotype calls (in VCF format) of 635 samples (release v7, hg19) from dbGaP. Indels and multi-allelic SNPs were removed using `bcftools` and variants with missing rate large than 10% were filtered out. We performed phasing on all 635 samples, with the same reference panel and command used when phasing Geuvadis data and used the resulting output file to create a list of heterozygous SNPs for each individual. Then we applied the same approaches as for Geuvadis data to collect Total Read Count (TReC) and Allele-Specific Read Count (ASReC) per gene and per sample for TReCASE and RASQUAL.

We filtered out genes whose 75 percentile of gene expression is less than 20 or maximum gene expression is more than 25 million. The resulting 16,675 genes were included in the analysis. The total number of reads mapped to genes in whole blood samples vary from 1 to 30 million with fraction of allele-specific reads from 2.3% to 11.73%, with one apparent outlier (Sample ID: YEC3 labeled in red in Fig A3), and it was removed in the following analysis.

Covariates data including genotyping principal components, age, and gender was downloaded from `https://gtexportal.org/home/datasets`. 354 samples, having RNA-seq data from whole blood, genotype data and covariates data, were included

in the following eQTL mapping.

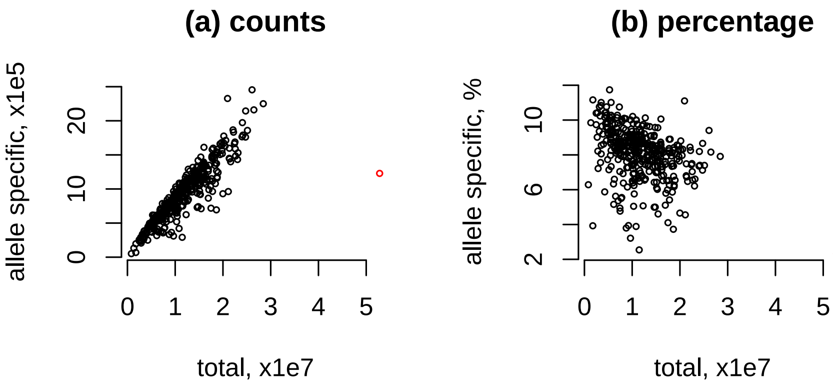

Fig. A3: Summary of total read counts (x-axis) versus total number of allele-specific reads (panel (a)) or the percentage of reads being allele-specific (panel (b)) across all genes per sample for GTEx data. The red point indicates sample YEC3 has unexpected low proportion of allele-specific reads and it is excluded from further analysis.

A.3 GTEx v8 dataset

A.3.1 Genotype and covariates data

Using the GTEx v8 release, we conducted eQTL analyses for 22 GTEx tissues with sample size larger than 300 together with an additional artery tissue of artery-coronary, and 5 brain tissues: Caudate (basal ganglia), Cerebellar Hemisphere, Cortex, Frontal Cortex, and Nucleus accumbens (basal ganglia). We used the same set of covariates as GTEx [13] in eQTL analysis. The covariate data were downloaded from GTEx data portal: file `GTEx_Analysis_v8_eQTL_covariates.tar.gz` from `https://gtexportal.org/home/datasets`. The covariates include genotype principal components, PEER factors derived from gene expression data, and a few other variables such as sex, pcr, and platform. Following the GTEx study, we did not use age as a covariate for eQTL mapping, but we used age in dynamic eQTL studies. The age information were obtained from file `GTEx_Analysis_v8_Annotations_`

SubjectPhenotypesDS.txt on GTEx data portal.

The genotype calls from whole genome sequencing were part of the GTEx V8 protected data, that are available at https://anvil.terra.bio/#workspaces/anvil-datastorage/AnVIL_GTEx_V8_hg38. Access of such data require approved access to the GTEx data on dbGaP. The exact file we used is: GTEx_Analysis_2017-06-05_v8_WGS_VCF_files_GTEx_Analysis_2017-06-05_v8_WholeGenomeSeq_838Indiv_Analysis_Freeze.SHAPEIT2_phased.vcf. Because the genotype data were from whole genome sequencing, there is no need for further imputation, and it is already phased. Therefore, we just directly extract the list of heterozygous SNPs for each individual and use this list to calculate ASE.

A.3.2 Processing RNA-seq on a cloud computing platform

The Geuvadis dataset has manageable size so that it can be downloaded and processed in a local computational environment, either a desktop or a computer cluster. In GTEx analysis, we seek to reanalyze the data from 28 tissues. For the GTEx dataset and possibly many other datasets, the raw data are too large to download to process locally. We developed a pipeline for data processing using the data stored in cloud. Although this pipeline was designed for the GTEx data saved in NHGRI AnVIL https://anvil.terra.bio/, it can be applied to other datasets as well. This pipeline is available at a GitHub repository, gtex_AnVIL: https://github.com/Sun-lab/gtex_AnVIL.

This pipeline processes the RNA-seq data sample by sample. For each sample, the input are raw RNA-seq data (e.g., bam files), a list of phased heterozygous SNPs, and annotation of genes, e.g., the transcript/exon annotations from GECODE [14]. The output is a text file with 4 columns of counts that correspond to total counts and counts haplotype 1 (hap1), haplotype 2 (hap2), and haplotype N (hapN). The definitions of hap1, hap2, and hapN are the same as those defined for the Geuvadis dataset. There are also row names that correspond to gene symbol. An example is shown below.

```
sample1.bam sample1_hap1.bam sample1_hap2.bam sample1_hapN.bam
ENSG00000000003.14 9 0 0 0
ENSG00000000005.5 0 0 0 0
ENSG00000000419.12 209 0 0 0
ENSG00000000457.13 303 51 40 0
ENSG00000000460.16 49 2 1 0
ENSG00000000938.12 29290 58 42 0
ENSG00000000971.15 46 1 8 0
ENSG00000001036.13 262 32 30 0
```

The pipeline is written by Workflow Description Language (WDL) (`collect_TReC_ASReC.wdl` in gtex_AnVIL repository), which calls computational engine embedded in a Docker file (folder `Docker` in gtex_AnVIL repository). An R script within the docker performed quality controls select RNA-seq reads with average base quality ≥ 20, mapping quality ≥ 20 and additional filtering provided by on R package `Rsamtools`:

```
flag1  = scanBamFlag(isUnmappedQuery=FALSE, isSecondaryAlignment=FALSE,
                     isDuplicate=FALSE, isNotPassingQualityControls=FALSE,
                     isSupplementaryAlignment=FALSE, isProperPair=TRUE)

param1 = ScanBamParam(flag=flag1, what="seq", mapqFilter=255)
```

We further filter genes for eQTL mapping and only consider the genes that have at least 10 total counts in at least 20% of the samples. We consider ASE of a gene if it has at least 5 allele-specific counts in 5 or more samples.

B Supplementary Methods

B.1 Probability distributions used by TReCASE and RASQUAL

Because we perform eQTL analysis for each gene separately, in the following section we define the probability distribution for one gene and omit the index for gene.

B.1.1 TReCASE definition

Let T_i be the total read count (TReC) for a gene of interest in the i-th sample, with $i = 1, \ldots, n$. In TReCASE model, T_i is assumed to follow a negative binomial distribution with density function defined as:

$$f_{NB}(T_i = t_i; \mu_i, \phi) = \frac{\Gamma(t_i + 1/\phi)}{\Gamma(t_i + 1)\Gamma(1/\phi)} \left(\frac{1/\phi}{1/\phi + \mu_i}\right)^{1/\phi} \left(\frac{\mu_i}{1/\phi + \mu_i}\right)^{t_i}, \tag{1}$$

where μ_i is sample-specific mean value and ϕ is an over-dispersion parameter. It can be show that the variance of T_i is

$$\mathrm{Var}(T_i) = \mu_i + \mu_i^2 \phi, \tag{2}$$

and in the limiting case when $\phi \to 0$, the negative binomial distribution converges to a Poisson distribution.

Sample-specific mean value μ_i is defined as a function of some covariates:

$$\log(\mu_i) = \beta_0 + \beta_\kappa \log(\kappa_i) + \sum_{u=1}^{p} \beta_u c_{iu} + \eta_i, \tag{3}$$

where β_0 is an intercept, β_κ is the coefficient for log-read-depth $\log(\kappa_i)$, β_u, $u = 1, ..., p$, is the regression coefficient for the u-th covariate (e.g., age, gender, batch effects etc.), and η_i is the genetic effect. Given a candidate eQTL with two alleles A and B, let g_i be its genotype, defined as the number of B alleles such that $g_i = 0$, 1, or 2 for genotype of homozygous A allele, heterozygous, or homozygous B allele, respectively. Then η_i is:

$$\eta_i = \begin{cases} 0 & \text{if } g_i = 0 \\ \log[1 + \exp(b_0)] & \text{if } g_i = 1 \\ b_0 & \text{if } g_i = 2 \end{cases}, \tag{4}$$

where b_0 is the genetic effect, defined as log ratio of gene expression for genotype BB vs. AA. The derivation of η_i follows from the assumption that gene expression is additive across the two alleles before log-transformation, see equations (3)-(7) of [9] for details.

For TReCASE, allele-specific expression (ASE), or allele-specific read counts (ASReCs) are collected for two haplotypes at gene-level, whereas the ASReCs for RASQUAL are measured at SNP level, as described in the next section. For the i-th sample, denote the two ASReCs for haplotypes 1 and 2 by N_{i1} and N_{i2}, so that total ASReC for the i-th sample is $N_i = N_{i1} + N_{i2}$. Note that for each sample, which haplotype is defined as haplotype 1 is arbitrarily decided. The distribution of N_{i1} given N_i can be modeled by a beta-binomial distribution:

$$f_{BB}(N_{i1} = n_{i1}; N_i = n_i, \alpha_i, \beta_i) = \binom{n_i}{n_{i1}} \frac{\Gamma(n_{i1} + \alpha_i)\Gamma(n_i - n_{i1} + \beta_i)}{\Gamma(n_i + \alpha_i + \beta_i)} \frac{\Gamma(\alpha_i + \beta_i)}{\Gamma(\alpha_i)\Gamma(\beta_i)}, \quad (5)$$

where α_i and β_i are sample specific parameters and they are connected with expected proportion of reads of haplotype 1 (denoted by π_i) and over-dispersion (denoted by θ) of this beta-binomial distribution by

$$\pi_i = \frac{\alpha_i}{\alpha_i + \beta_i} \text{ and } \theta = \frac{1}{\alpha_i + \beta_i}. \quad (6)$$

The variance of this beta-binomial distribution is

$$\text{Var}(N_{i1}) = n_i \pi_i (1 - \pi_i) \frac{1 + n_i \theta}{1 + \theta},$$

which converges to the variance of binomial distribution when $\theta = 0$.

In an alternative parametrization, the over-dispersion parameter can be rescaled to interval [0,1): $\rho = \theta/(1 + \theta)$ [15]. We will switch to this parametrization in section C.6.4 to study inflation introduced by splitting reads across several SNPs within a gene.

Let G_i be the ordered genotype of the candidate eQTL, which takes values 0, 1, 2, and 3 for ordered genotype AA, AB, BA, and BB. An ordered genotype is

defined based on the order of haplotype 1 followed by haplotype 2. For example, AB indicates that A is on haplotype 1 and B is on haplotype 2. Given G_i, we can model π_i as a function of genetic effect b_0:

$$\log\left(\frac{\pi_i}{1-\pi_i}\right) = \begin{cases} -b_0 & \text{if } G_i = AB \\ b_0 & \text{if } G_i = BA \\ 0 & \text{if } G_i = AA \text{ or } BB \end{cases}. \quad (7)$$

The genetic effect b_0 for TReC (equation (4)) and ASE (equation (7)) are the same and thus it can be estimated by combining the data from TReC and ASE.

B.1.2 RASQUAL definition

To illustrate the difference between TReCASE and RASQUAL, we only consider the basic RASQUAL model without additional features such as capturing sequencing/mapping error or reference allele mapping bias. In addition, we change the notations used by RASQUAL to be consistent with the notations of TReC to facilitate comparison. We will adopt two terms used in the RASQUAL paper: *cis*-regulatory SNP (**rSNP**) and feature SNP (**fSNP**). An rSNP is a candidate eQTL SNP and a fSNP is a SNP within exonic region of a gene where ASReC can be measured.

RASQUAL also models TReC T_i by a negative binomial distribution. Though instead of a common over-dispersion parameter for all samples, it has a sample-specific over-dispersion parameter ϕ_i:

$$f_{NB}(T_i = t_i; \mu_i, \phi_i) = \frac{\Gamma(t_i + 1/\phi_i)}{\Gamma(t_i+1)\Gamma(1/\phi_i)}\left(\frac{1/\phi_i}{1/\phi_i + \mu_i}\right)^{1/\phi_i}\left(\frac{\mu_i}{1/\phi_i + \mu_i}\right)^{t_i}. \quad (8)$$

The mean and over-dispersion is parametrized by

$$\mu_i = \lambda K_i Q_i \text{ and } \phi_i = \frac{1}{\theta_R K_i Q_i}, \quad (9)$$

where λ is a scaling parameter for absolute mean of coverage depth of this gene, K_i is a sample specific offset reflecting library size and other a priori estimated size factors, Q_i is genetic effect defined later, and θ_R is a scaling parameter for over-dispersion.

Then the variance of T_i is $\text{Var}(T_i) = \mu_i + \mu_i^2\phi_i = \mu_i(1 + \lambda/\theta_R)$.

Recall that g_i denotes the genotype of a candidate eQTL, and it equals to 0, 1, or 2 for genotype AA, AB/BA, or BB. RASQUAL quantifies genotype effect by

$$Q_i = \begin{cases} 2(1-\pi) & \text{if } g_i = 0, \\ 1 & \text{if } g_i = 1, \\ 2\pi & \text{if } g_i = 2. \end{cases}$$

Then the sample-specific mean value in log scale is

$$\log(\mu_i) = \log(\lambda K_i Q_i) = \log(\lambda) + \log(K_i) + \log(Q_i). \tag{10}$$

Comparing the mean value of TReCASE (equation (3)) versus RASQUAL (equation (10)), it is easy to see that $\log(K_i)$ captures the effects of all the covariates. Although it is not explicitly mentioned in the RASQUAL paper, we expect the scale of K_i to be around 1 because they assume λ captures the absolute mean value μ_i. We can also see the correspondence of genetic effect between TReCASE (b_0) and RASQUAL (π) is $\log[\pi/(1-\pi)] = b_0$.

While there is minor difference between TReCASE and RASQUAL in their TReC model, the major difference is their specifications for the ASE data. In RASQUAL, ASReCs are measured for each feature SNP (fSNP) (with SNP index $l = 1, \ldots, L$), denoted by N_{il0} and N_{il1} (with $N_{il} = N_{il0} + N_{il1}$). Then given N_{il}, N_{il1} is modeled by a beta-binomial distribution

$$f_{BB}(N_{il1} = n_{il1}; N_{il} = n_{il}, \alpha_{il}, \beta_{il}) = \binom{n_{il}}{n_{il1}} \frac{\Gamma(n_{il} - n_{il1} + \alpha_{il})\Gamma(n_{il1} + \beta_{il})}{\Gamma(n_{il} + \alpha_{il} + \beta_{il})} \frac{\Gamma(\alpha_{il} + \beta_{il})}{\Gamma(\alpha_{il})\Gamma(\beta_{il})}. \tag{11}$$

Let $0 < h \leq 1/L$ be the relative proportion ASReC contributed by each fSNP, then

$$\alpha_{il} = h\theta_R K_i Q_{il0} \text{ and } \beta_{il} = h\theta_R K_i Q_{il1},$$

where Q_{il0} and Q_{il1} quantify the number of ASReC from haplotype 0 and haplotype 1, and they are defined in Supplementary Table 4 of RASQUAL paper [16]. Note

that $Q_{il} = Q_{il0} + Q_{il1} = Q_i$, which only depends on the genotype of the *cis*-regulatory SNP (rSNP, or candidate eQTL) but not the genotype of the l-th fSNP.

Then the expected proportion of reads of haplotype 1 and over-dispersion (denoted by ϑ) of this beta-binomial distribution are

$$\pi_i = \frac{\alpha_i}{\alpha_i + \beta_i} = \frac{Q_{il1}}{Q_i} \text{ and } \vartheta = \frac{1}{\alpha_i + \beta_i} = \frac{1}{h\theta_R K_i Q_i}. \quad (12)$$

The variance of this beta-binomial distribution is

$$\text{Var}(N_{il1}) = n_{il1}\pi_i(1-\pi_i)\frac{1+n_i\vartheta}{1+\vartheta},$$

which converges to the variance of binomial distribution when $\vartheta = 0$.

In the RASQUAL paper, the authors further set $h = 1$ for the following reasons quoted from page 42 of their supplementary materials:

> "Here the constant h reflecting the proportion of total AS count at each feature SNP is arbitrary ($0 < h \leq 1/L$ for $L > 0$; otherwise $h = 0$). However, in our experience, $h < 1/L$ usually gives worse result in terms of power and fine-mapping than $h \approx 1$. This is partly because the larger the number of feature SNPs L is, the smaller the proportion h each feature SNP accounts for, resulting in overestimation of the dispersion parameter $\hat{\theta}$, resulting in more significant associations in hypothesis testing for features with larger L. To avoid this issue we set $h = 1$ to penalize the over-dispersion parameter more for large L."

Since h denotes the proportion ASReC from each fSNP, it counter-intuitive to set it to be 1. It appears to be an ad-hoc solution to reduce the significance level, especially for the genes with large number of fSNPs. In fact, as shown in this paper, even when $h = 1$, RASQUAL still has inflated type I error, and the degree of inflation increases with the number of fSNPs (Fig 5B in main text). However, the reason is not overestimation of the dispersion parameter $\hat{\theta}$. The estimate of the dispersion parameter is often accurate. It is the mis-specified likelihood model that leads to underestimate of the variance of the eQTL effect size.

B.1.3 Definition of RASQUAL-like method: TReCASE-RL

To facilitate more pointed comparison between TReCASE and RASQUAL, in addition to running RASQUAL, we also implemented a modification of TReCASE to adopt two key assumptions made by RASQUAL but not by TReCASE: (1) Equating the over-dispersion parameters of the negative binomial distribution for TReC and the beta-binomial distribution for ASE; (2) Treating ASReC of each fSNP as independent beta-binomial observation. Specifically, for the i-th sample and the l-th SNP, we denote the two ASReCs for haplotypes 1 and 2 by N_{il1} and N_{il2}. The distribution of N_{il1} given $N_{il} = N_{il1}+N_{il2}$ is modeled by a beta-binomial distribution.

$$\begin{aligned} & f_{BB}(N_{il1} = n_{il1}; N_{il} = n_{il}, \alpha_{il}, \beta_{il}) \\ & \quad = \binom{n_{il}}{n_{il1}} \frac{\Gamma(n_{il1} + \alpha_{il})\Gamma(n_{il} - n_{il1} + \beta_{il})}{\Gamma(n_{il} + \alpha_{il} + \beta_{il})} \frac{\Gamma(\alpha_{il} + \beta_{il})}{\Gamma(\alpha_{il})\Gamma(\beta_{il})}, \end{aligned} \quad (13)$$

where α_{il} and β_{il} are SNP specific parameters connected with expected proportion of reads of haplotype 1 (denoted by π_i) and over-dispersion (denoted by θ) of this beta-binomial distribution by

$$\pi_{il} = \frac{\alpha_{il}}{\alpha_{il} + \beta_{il}} \text{ and } \theta = \frac{1}{\alpha_{il} + \beta_{il}}. \quad (14)$$

We call the modified TReCASE model as TReCASE-RL, where RL stands for "RASQUAL Like". By comparing TReCASE and TReCASE-RL, we can illustrate the consequence of these two assumptions.

B.1.4 The over-dispersion parameters of the three models

We summarize the over-dispersion parameters of three models: TReCASE, RASQUAL, and TReCASE-RL, in Table A1. The over-dispersion parameters of TReCASE are constants across all samples. In contrast, RASQUAL's over-dispersion parameters vary across samples because they depend on K_i and Q_i. We expect both K_i and Q_i vary around the value of 1 and thus we may approximate the over-dispersion parameters of RASQUAL by assuming $K_i = Q_i = 1$. Then we can see the definition of over-dispersion parameters are in reverse scale. For TReCASE, larger over-dispersion parameter (ϕ or θ) means larger over-dispersion, and for RASQUAL, larger over-dispersion parameter (θ_R) means smaller over-dispersion. In our results, when we refer to an over-dispersion, it is the TReCASE over-dispersion.

Table A1: Over-dispersion parameters in TReCASE, RASQUAL, and TReCASE-RL models. RASQUAL model modifies the degree of over-dispersion variation with additional offset K_i and relative genetic effect Q_i.

Component	TReCASE	RASQUAL	TReCASE-RL
TReC	ϕ	$1/(\theta_R K_i Q_i) \approx 1/\theta_R$	θ
ASE	θ	$1/(\theta_R K_i Q_i) \approx 1/\theta_R$	θ

B.2 Simulation setup

B.2.1 Simulation for TReC

We simulate total read count (TReC) using 4 covariates (including one that can be treated as library depth). Negative binomial over-dispersion parameter is set to be 0.01, 0.1 or 0.5. Genetic effect size b_0 is set to be 0, 0.125, 0.25, or 0.5. We simulate the TReC so that the median across samples is around 100.

B.2.2 Simulation for ASReCs without within-sample over-dispersion

In the basic simulation setup, we simulate ASReCs within a sample by a binomial distribution and introduce over-dispersion across samples. Specifically, we simulated data by the following steps:

1. We assume 10% of TReC are allele-specific (Fig A4(a)) and set the same genetic effect b_0 on ASReC as for TReC. The beta-binomial over-dispersion is defined as a fraction of negative binomial over-dispersion, with several values: 0, 1/8, 1/4, 1/2, 1 and 2 to cover the fractions observed in the Geuvadis data (Fig A4(b)). Fraction 0 corresponds to the extreme, but not unlikely scenario when ASReC follow a binomial distribution.

2. After calculating expected proportion of reads coming from allele B for each genotype G, denoted by π_G we generate sample level expected proportions π_i by sampling from a beta distribution with parameters α and β such that $\pi_G = \alpha/(\alpha + \beta)$, and $\theta = 1/(\alpha + \beta)$.

3. Given π_i, we generate ASReC from a binomial distribution.

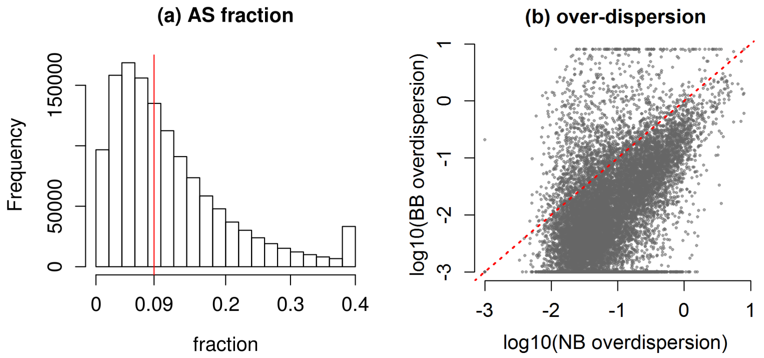

Fig. A4: (a) The fraction of RNA-seq reads being allele-specific per gene per sample, given the ASReC is larger than 0, and truncated at 0.4. The vertical line indicates median. It is based on Geuvadis dataset of 280 samples. (b) The distribution of over-dispersion parameters estimated by TReCASE from the Geuvadis dataset of 280 samples. The BB over-dispersion is truncated at 0.001.

To simulate SNP-level ASReC, we modify step 3 as follows:

3a Uniformly distribute allele-specific reads (that can belong to either haplotype) among 2, 4, or 8 SNPs, and then simulate SNP-level ASReC on one haplotype by a binomial distribution using the same sample level proportion π_i.

B.2.3 Add within-sample over-dispersion for ASReC data

In order to simulate SNP level ASReC with over-dispersion across multiple SNPs within a sample, we modify step 3a as follows:

3b Uniformly distribute the allele-specific reads (that can belong to either haplotype) among 2, 4 or 8 SNPs, and then simulate SNP-level ASReC for each haplotype by a beta-binomial distribution with mean value π_i and an over-dispersion parameter that equals to the between-sample beta-binomial over-dispersion.

This modification allows us to obtain ASReCs with over-dispersion within a sample and at the same time with more similarity within sample than between samples. Fig 5(c) in the main text use the results from this simulation setup.

B.2.4 To simulate RASQUAL style ASReC data

RASQUAL style SNP level ASReC data have the same over-dispersion across any two SNPs, either two SNPs within a sample or between samples. In other words, there is no extra over-dispersion across samples. To simulate such data, we modify step 2 to set the between-sample over-dispersion to be 0. After that we generate all SNP level ASReC from a beta-binomial distribution according to step 3b. This is equivalent to generating SNP level proportions π_{il} from sample level proportions π_i with the same over-dispersion θ without discrimination for the SNPs within and between samples.

B.2.5 Simulating the data with genotyping errors

To simulate genotyping errors, we randomly flip a fraction of genotypes from homozygous to heterozygous and from heterozygous to homozygous (fractions 0.05, 0.10 and 0.20 were used). The wrong genotypes have the following consequences.

1. TReCASE only uses heterozygous SNPs to collect ASReCs. RASQUAL also produces counts for homozygous SNPs. Consequently, whenever we flip a heterozygous SNP to be homozygous, the read counts of this SNP are ignored by TReCASE, while still used by RASQUAL - it would have read counts of both alleles counted, but attach them to an incorrect homozygous genotype status.

2. When a truly homozygous SNP is flipped to be heterozygous, both methods still use such data. They both assume all the reads are from one of the two alleles.

C Supplementary Results

C.1 Estimation of permutation p-value estimation using geoP

C.1.1 Evaluation of geoP using Geuvadis dataset

As a quick alternative to estimate permutation p-values, one might simply use eigenMT [17] to estimate the effective number of tests, and then obtain the permutation p-value estimate by multiplying minimum p-value with the effective number of tests and truncating at 1. We evaluate this approach and our methods when estimating the relation between permutation p-value and nominal p-value using a linear regression or a logistic regression. As a gold standard to compare with, we estimated permutation p-values estimated by 10,000 permutations for 14,566 genes.

EigenMT tends to be conservative with a large number of false negatives (permutation p-value estimated using EigenMT is larger than a threshold α while the estimates by 10,000 permutations is smaller than α), especially at less significant p-values (Table A2). This suggests that the eigenMT estimates of the number of tests is too large, particularly so for larger p-values. Overall the results based on linear fit is much more accurate than eigenMT (in terms of smaller number of false positives + false negatives), though it produces unbalanced false positives and false negatives, with more false positives at larger p-value cutoff and more false negatives at smaller p-value cutoffs (Table A2). Finally, the logistic regression has the the most accurate estimates with balanced numbers of false positives and false negatives (Table A2).

In terms of false classifications, we do not get as much improvement by using more than 100 grid points, particularly for logistic regression (glm) approach (Table A3). The performance of linear regression and logistic regression become similar at larger grid points and more significant p-values. Consequently, we suggest using 100 grid points to estimate permutation p-values, though even with 25 grid points we observe large improvement against eigenMT.

The estimation bias by eigenMT or linear model, as well as the advantage of using larger number of grid points, can be visualized by the scatter plots of permutation p-value estimates versus the "true values" estimated by 10,000 permutations

Table A2: Permutation p-value misclassification by method. Misclassification by three methods for 14,566 genes from Geuvadis dataset. Quantifying by false positives (f.pos), false negatives (f.neg) and overall number of mis-classification (f.pos + f.neg). Here a positive outcome means the permutation p-value estimate is smaller than the cutoff value α.

	eigenMT			lm			glm			total
α	f.neg	f.pos	all	f.neg	f.pos	all	f.neg	f.pos	all	n.pos
1e-1	1088	5	1193	7	97	104	27	18	45	6207
5e-2	694	4	871	5	85	90	34	25	59	5143
1e-2	275	10	285	21	36	57	17	30	47	3678
5e-3	219	12	207	31	24	55	17	30	47	3301
1e-3	85	25	110	52	26	78	28	39	67	2664

Table A3: Number of misclassifications of permutation p-value estimates for 25, 50, 100 or 200 grid points.

permutation	eigenMT	lm				glm			
p-value cutoff		25	50	100	200	25	50	100	200
0.1	1093	139	103	104	94	66	61	45	42
0.05	698	112	99	90	88	74	53	59	55
0.01	285	65	65	57	57	47	52	47	47
0.05	219	77	59	55	48	59	54	47	51
0.001	110	81	79	78	69	72	59	67	60

(Fig A5).

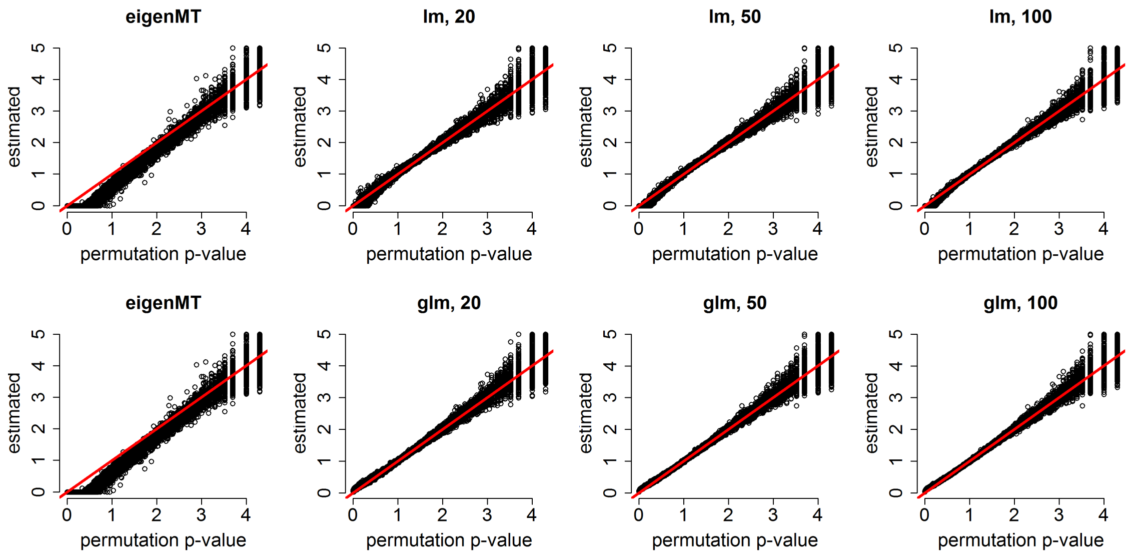

Fig. A5: Permutation p-value estimation using three methods for Geuvadis dataset: eigenMT, linear model (lm), and logistic model (glm). The number following lm or glm is the number of grid points used. The x-axis are permutation p-values estimated by 10,000 permutations.

C.1.2 Evaluation of geoP using GTEx v7 dataset

Next we carried out similar evaluations of permutation p-value estimation using GTEx v7 data from whole blood. The conclusion is the same as those from Geuvadis dataset: glm has better performance than lm, which is better than eigenMT (Table A4) and 100 grid points is a good balance for computational cost and accuracy (Table A5). Finally, the scatter plot of permutation p-value estimates versus "true values" estimated by 10,000 permutations illustrate that glm with 100 grid points has the best estimation accuracy among all the methods (Fig A6).

Table A4: Permutation p-value misclassification by method.

Misclassification by three methods for 16,637 genes using GTEx v7 whole blood data. Quantifying by false positives, false negatives and overall number of mis-classification

	eigenMT			lm			glm			total
α	f.neg	f.pos	all	f.neg	f.pos	all	f.neg	f.pos	all	n.pos
1e-1	1000	0	1000	11	115	126	33	24	57	5001
5e-2	595	0	595	5	92	97	17	28	45	3887
1e-2	204	0	204	20	21	41	18	24	42	2489
5e-3	131	3	134	24	17	41	18	27	45	2177
1e-3	54	11	65	44	21	65	29	29	58	1708

Table A5: Number of misclassifications of permutation p-value estimates for 25, 50, 100 and 200 point grid.

permutation	eigenMT	lm				glm			
p-value cutoff		25	50	100	200	25	50	100	200
0.1	1000	184	121	126	106	82	67	57	60
0.05	595	100	108	97	89	55	43	45	44
0.01	204	52	50	41	38	48	45	42	34
0.005	134	65	60	41	48	50	45	45	46
0.001	65	71	62	65	57	55	56	58	54

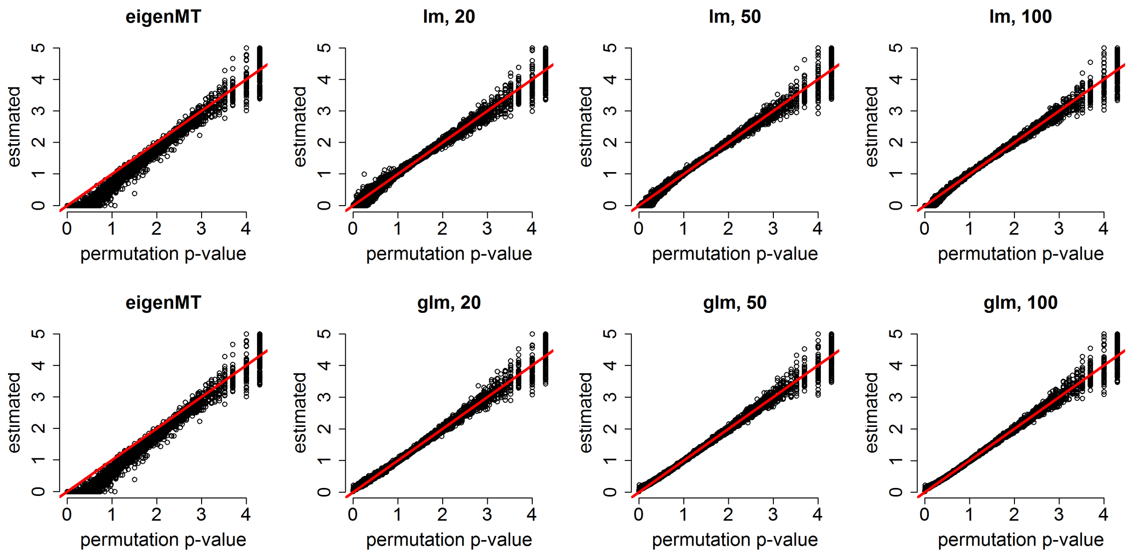

Fig. A6: Permutation p-value estimation using three methods: eigenMT, linear regression and logistic regression using GTEx v7 whole blood dataset. The x-axis are permutation p-values estimated by 10,000 permutations.

C.2 Use simulations to compare the power of TReCASE vs. MatrixEQTL.

We simulated count data according to TReCASE style assumption, i.e. without within sample over-dispersion for allele-specific counts across multiple heterozygous SNPs. This assumption is supported by the observation from real data, see sections C.5 for more details. We selected various sample sizes covering most of the sample size ranges in the GTEx datasets with additional cases of small sample sizes: starting from 32 samples to 512 samples with a step of 32 samples. We simulated 10,000 replicates for each setup with expected total count per gene being 100, of which 10% are allele-specific counts. We used several effect sizes corresponding to log fold change: 0, 0.2, 0.4 and 0.6 – as typical effect sizes for for real GTEx tissues, as illustrated in panel (a) of Fig A7. The over-dispersion parameters of both total count and allele-specific counts were set as 0.5. We fitted total and allele-specific counts using TReCASE model and the log transformed total counts, and then fitted linear regression using MatrixEQTL.

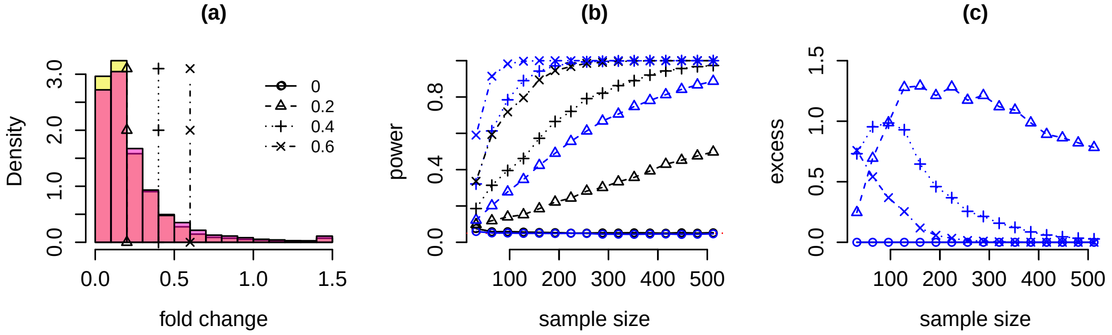

Fig. A7: Increase in power according to TReCASE vs linear model fit depends on effect size and sample size. (a) The distributions of eQTL effect sizes (log fold change) in two GTEx tissues - Brain Caudete basal ganglia (bright yellow) and Whole Blood (pink). The two histograms were overlaid on each other. Three vertical lines indicate three effect sizes used in our simulation. (b) Type I error (when log fold change is 0) and power (when log fold change is larger than 0) with respect to sample size. The shape of each point indicates effect size (see labels in panel (a)). The blue and black color indicate results from TReCASE and linear model, respectively. (c) The excess proportion of findings by TReCASE vs. Matrix eQTL.

The excess of power of TReCASE versus MatrixEQTL (i.e., the additional number of eQTLs identified by TReCASE divided by the total number of findings by MatrixEQTL) depends on eQTL effect size and sample size (Supplementary Fig A7 (c)). For log fold change of 0.2, which is a typical effect size in GTEx study (Fig A7 (a)) the power gain of TReCASE increase with sample size and reaches more than 120% power gain for sample size 150 to 300. For larger effect sizes, the power gain of TReCASE is more apparent for smaller sample sizes, e.g., below 200 for log fold change 0.5 and below 100 for log fold change of 0.8.

C.3 Supplementary results for GTEx v8 data.

C.3.1 Results of gene-SNP pairs

The following two figures illustrate the number of (gene, SNP) pairs with permutation p-value smaller than 0.01. We used the following tissue name abbreviations: Muscle Skeletal (MS), Whole Blood (WB), Skin Sun Exposed Lower leg (SSELI), Artery Tibial (AT), Adipose Subcutaneous (AS), Thyroid (Th), Nerve Tibial (NT), Skin Not Sun Exposed Suprapubic (SNSES), Lung (L), Esophagus Mucosa (EMuc), Cells Cultured fibroblasts (CCf), Adipose Visceral Omentum (AVO), Esophagus Muscularis (EMus), Breast Mammary Tissue (BMT), Artery Aorta (AA), Heart Left Ventricle (HLV), Heart Atrial Appendage (HAA), Colon Transverse (CT), Esophagus Gastroesophageal Junction (EGJ), Stomach (S), Testis (Te), Pancreas (P), Artery Coronary (AC), Brain Cortex (BC), Brain Nucleus accumbens basal ganglia (BNabg), Brain Caudate basal ganglia (BCbg), Brain Cerebellar Hemisphere (BCH), and Brain Frontal Cortex BA9 (BFC).

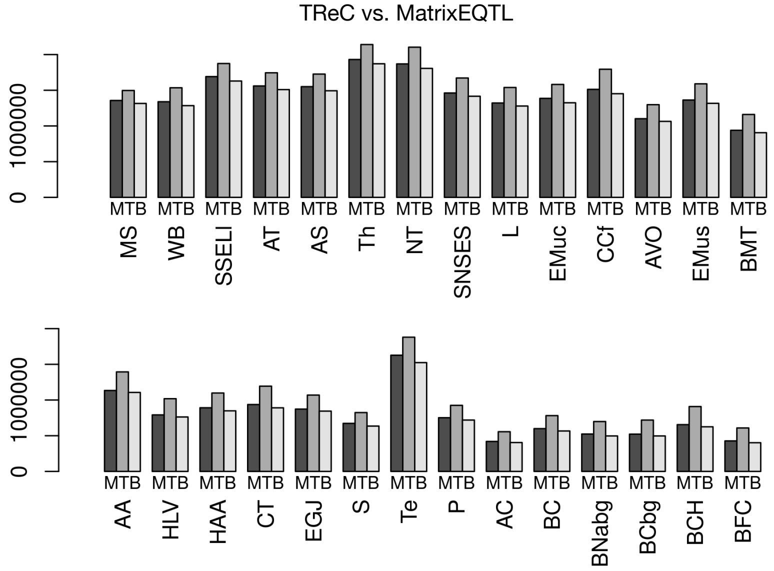

Fig. A8: The number of gene-SNP eQTL pairs identified by MatrixEQTL (M), TReC (T) or both (B) methods.

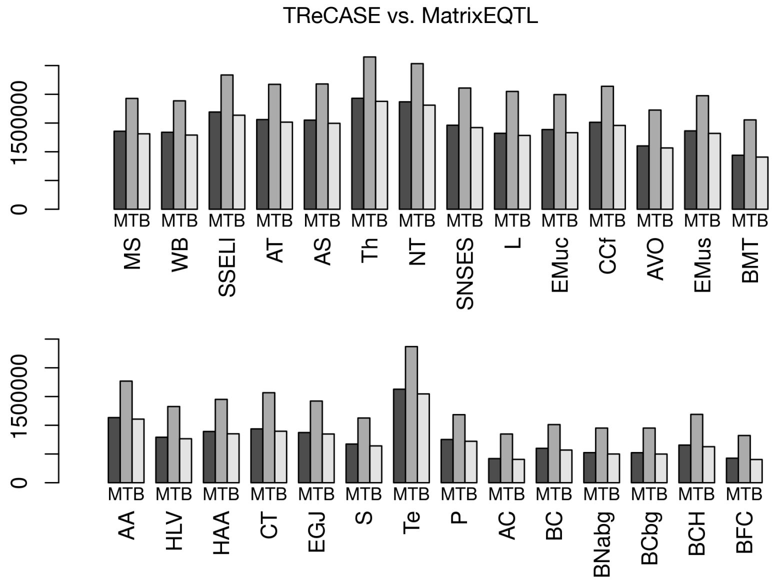

Fig. A9: The number of gene-SNP eQTL pairs identified by MatrixEQTL (M), TReCASE (T) or both (B) methods.

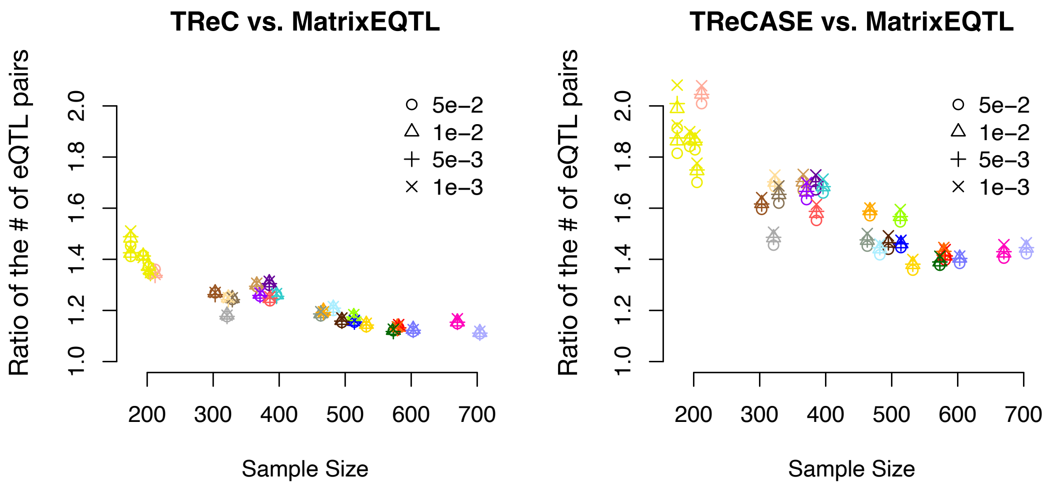

Fig. A10: The ratio of the number of eQTL pairs identified by TReC or TReCASE over the number of eQTL pairs identified by MatrixEQTL at different permutation p-value cutoffs (indicated by point labels) for different tissues (indicated by colors). Here the color scheme is the same as the colors used in Fig 2 of main text.

C.3.2 Examples of the eQTLs missed by MatrixEQTL but captured by TReCASE

Here we illustrate four eQTLs (two from whole blood and two from brain Caudate basal ganglia) that are missed by MatrixEQTL but captured by TReCASE.

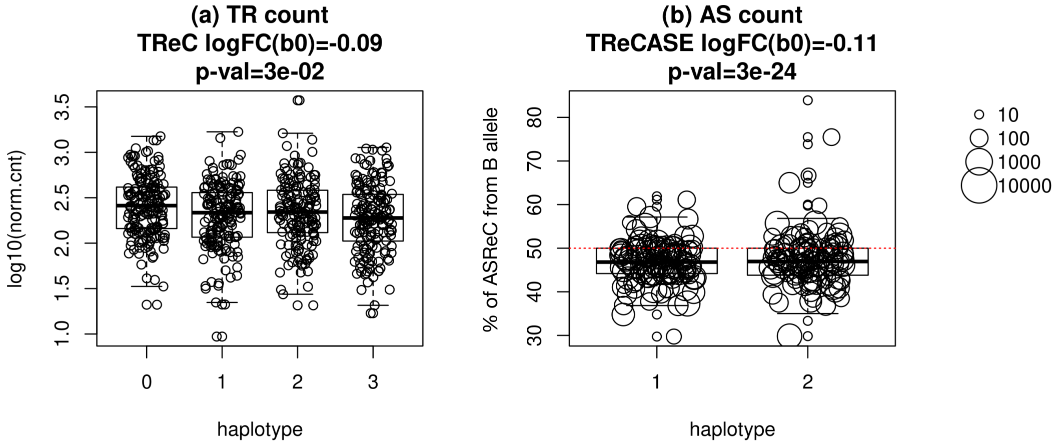

Fig. A11: An example for gene *SPARC* (ENSG00000113140)'s expression in whole blood. Panel (a) illustrates read-depth corrected total expression in log10 scale for four types of haplotype configurations that connect the eQTL (with alleles A and B) with the two haplotype of the eGene: haplotype 1 (h1) and haplotype 2 (h2). 0: A-h1, A-h2, 1: A-h1, B-h2, 2: A-h2, B-h1, 3, B-h1, B-h2. In other words, 0 and 3 indicate two homozygous genotypes and 1 and 2 indicate two ordered heterozygous genotypes. Panel (b) illustrates the percentage of allele-specific counts coming from the haplotype associated with the B allele of the eQTL. For example, for haplotype configuration 1 (A-h1 and B-h2), it is the proportion of counts from haplotype h2, and for haplotype configuration 2 (A-h2 and B-h1), it is the proportion of counts from haplotype h1. The size of the circles in panel (b) indicates the number of allele-specific reads. The subtitle of panel (a) and (b) provides the log fold change and permutation p-value for TReC model and TReCASE model, respectively.

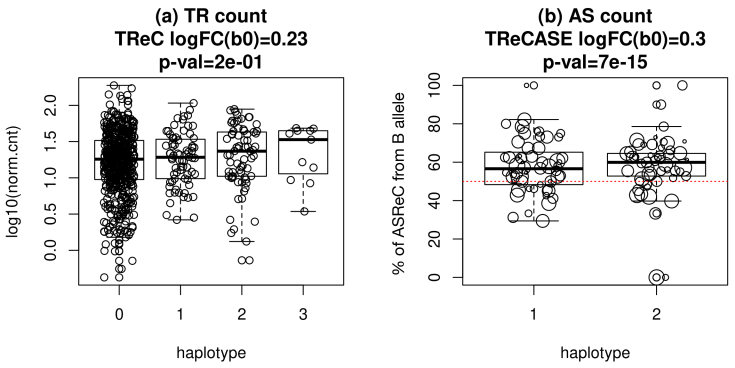

Fig. A12: An example for gene *IL1RL1* (ENSG00000115602)'s expression in whole blood.

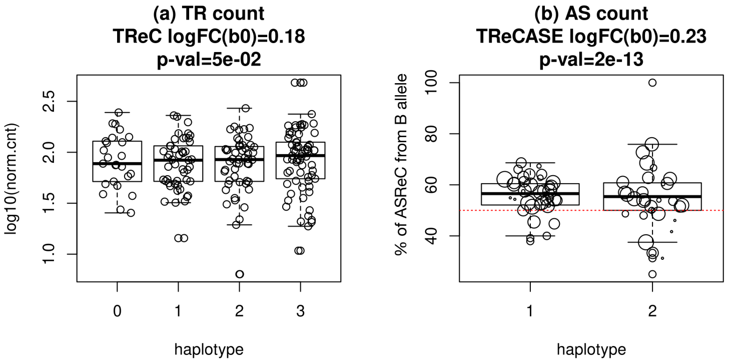

Fig. A13: An example for gene *TIE1* (ENSG00000066056)'s expression in brain Caudate basal ganglia.

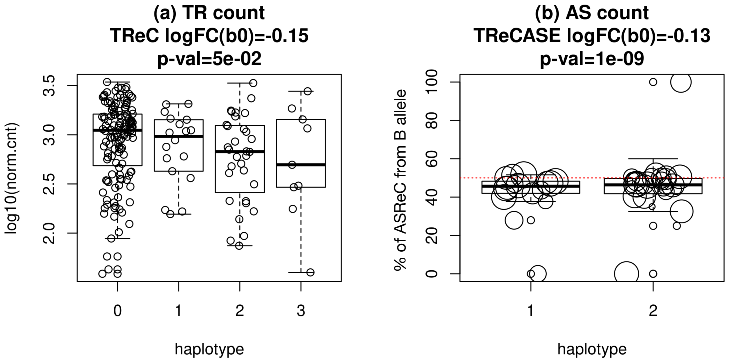

Fig. A14: An example for gene *NGEF* (ENSG00000066248)'s expression in brain Caudate basal ganglia.

C.3.3 GWAS enrichment

Our GWAS enrichment analyses were motivated by GTEx's approach [13] to obtain jackknife-based estimates and confidence intervals. Here we provide formal notations for the calculations. First order all gene-SNP pairings by their genomic locations (first by the location of the gene, and then by the location of the SNP). Let E_l, G_l, and T_l denote binary indicators for whether or not the lth gene-SNP pair is considered an eQTL, the locus is considered a GWAS hit (with respect to a phenotype group or simply contained in the GWAS catalog of all phenotypes), and the locus was tested in our analysis, respectively. Log-transformed enrichment, denoted by R, is then defined as

$$R = \log\left[\frac{\sum_l E_l G_l T_l}{\sum_l G_l T_l} / \frac{\sum_l E_l T_l}{\sum_l T_l}\right].$$

To perform the block jackknife procedure, divide the ordered gene-SNP pairs into B separate and approximately equal-sized blocks. For $b = 1, \dots, B$, calculate enrichment leaving out the bth block of gene-SNP pairs. Let $C_{lb} = 1$ indicate that the lth gene-SNP pair is contained in the bth block and $C_{lb} = 0$ otherwise. Then the leave-one-block-out log-transformed enrichment estimate is

$$R_b = \log\left[\frac{\sum_l (1 - C_{lb}) E_l G_l T_l}{\sum_l (1 - C_{lb} G_l T_l} / \frac{\sum_l (1 - C_{lb}) E_l T_l}{\sum_l (1 - C_{lb}) T_l}\right].$$

Define $\tilde{R}_b = R + (B-1)(R - R_b)$ as the bias-corrected log enrichment after excluding the bth block. Finally, we calculate jackknife-based mean and variance estimate of log enrichment as

$$\tilde{R} = \frac{1}{B}\sum_b \tilde{R}_b \text{ and } V_R = \frac{1}{B-1}\sum_b \left(\tilde{R}_b - \tilde{R}\right)^2,$$

respectively. The jackknife 95% confidence interval for log enrichment is

$$\tilde{R} \pm 1.96\sqrt{\frac{1}{B} V_R}.$$

If there is no overlap between eQTLs and GWAS hits across all gene-SNP pairs ($\sum_l E_l G_l T_l = 0$ or $\sum_l (1 - C_{bl}) E_l G_l T_l = 0$), the overall and jackknife log enrichment estimates and confidence intervals are set to zero. If the number of overlaps between

eQTLs and GWAS is small (e.g. < 5), this is reflected by larger confidence intervals. This procedure is performed per tissue type and model, treating G_l as any GWAS hit across phenotypes (GWAS catalog) or specific to a phenotype. In Supplementary Fig A15, we provide R as black dots, $\tilde{R}$ as open or filled circles as well as bars for confidence intervals, indicating statistically significant or insignificant enrichment.

Fig. A15: Enrichment of GWAS hits among eQTLs of 28 GTEx tissues identified by OLS (odinary least square, i.e., liner regression by MatrixEQTL) or TReCASE. The results of 28 GTEx tissues are split into 7 panels. The black dot is the point estimate of the log enrichment. The colored circle and intervals are bootstrap estimate and confidence interval. The solid circles are significant findings. Here we only consider the results are significant if the enrichment is positive, i.e., if eQTLs are more likely to be GWAS than non-eQTLs.

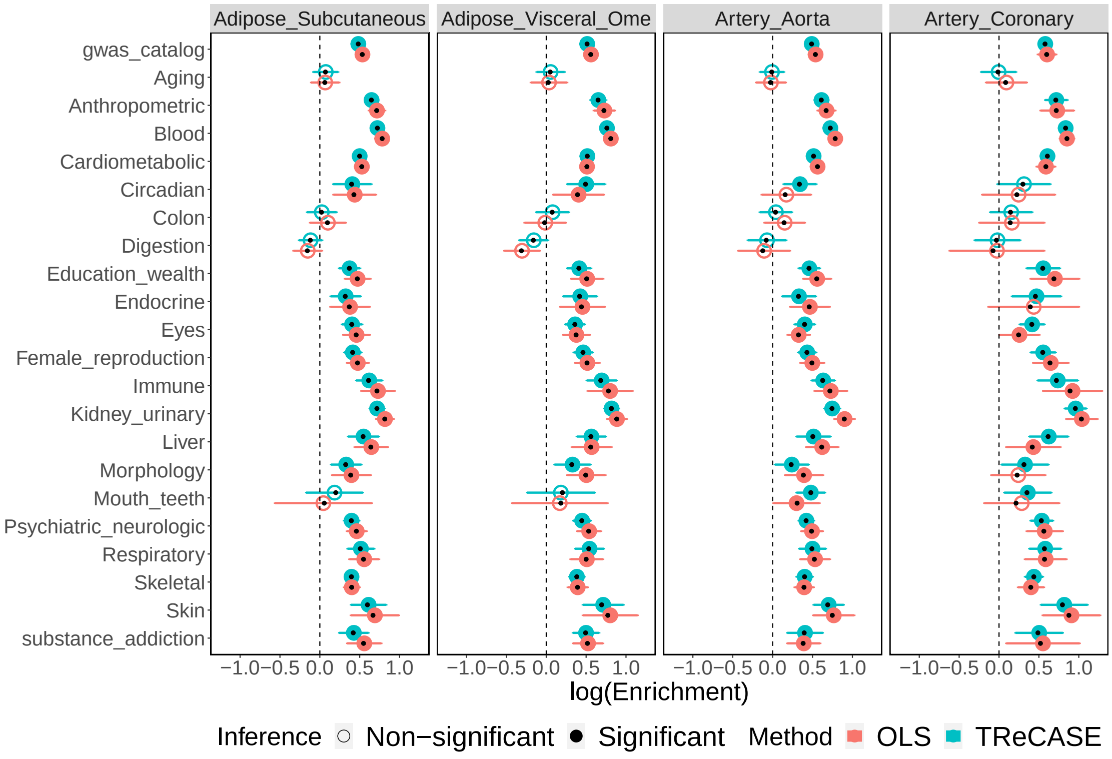

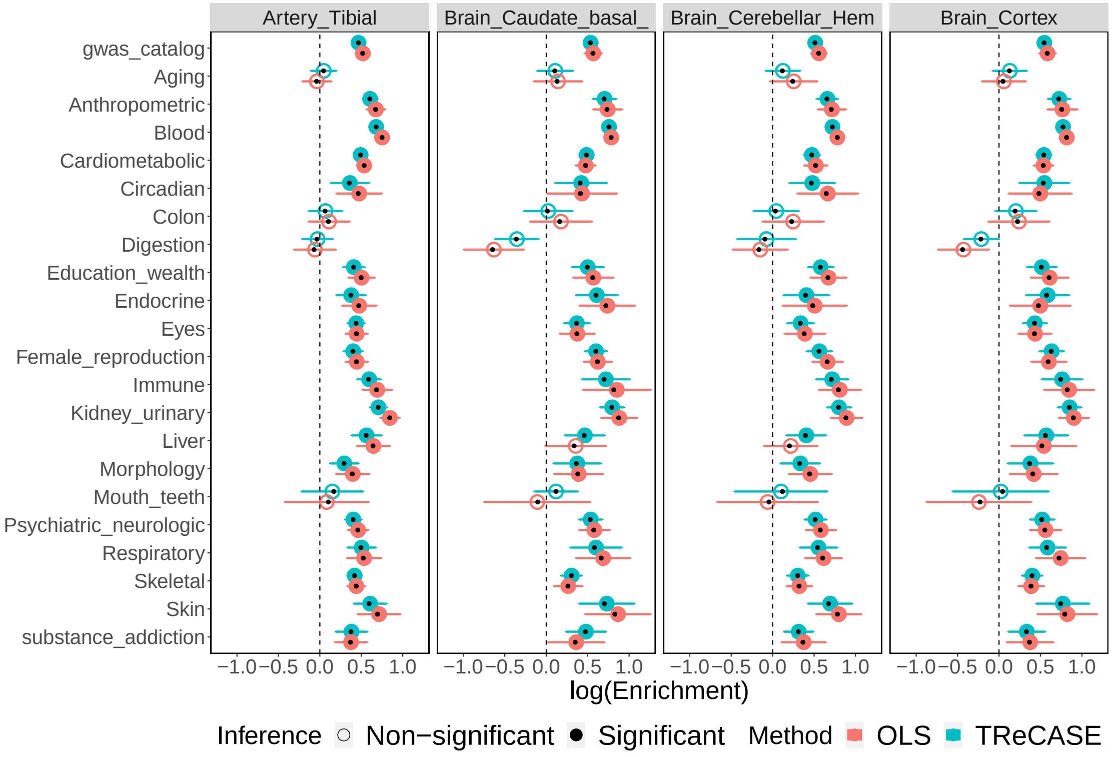

Artery_Tibial
Brain_Caudate_basal_
Brain_Cerebellar_Hem
Brain_Cortex
gwas_catalog
Aging
Anthropometric
Blood
Cardiometabolic
Circadian
Colon
Digestion
Education_wealth
Endocrine
Eyes
Female_reproduction
Immune
Kidney_urinary
Liver
Morphology
Mouth_teeth
Psychiatric_neurologic
Respiratory
Skeletal
Skin
substance_addiction
−1.0 −0.5 0.0 0.5 1.0
log(Enrichment)
Inference Non−significant Significant Method OLS TReCASE

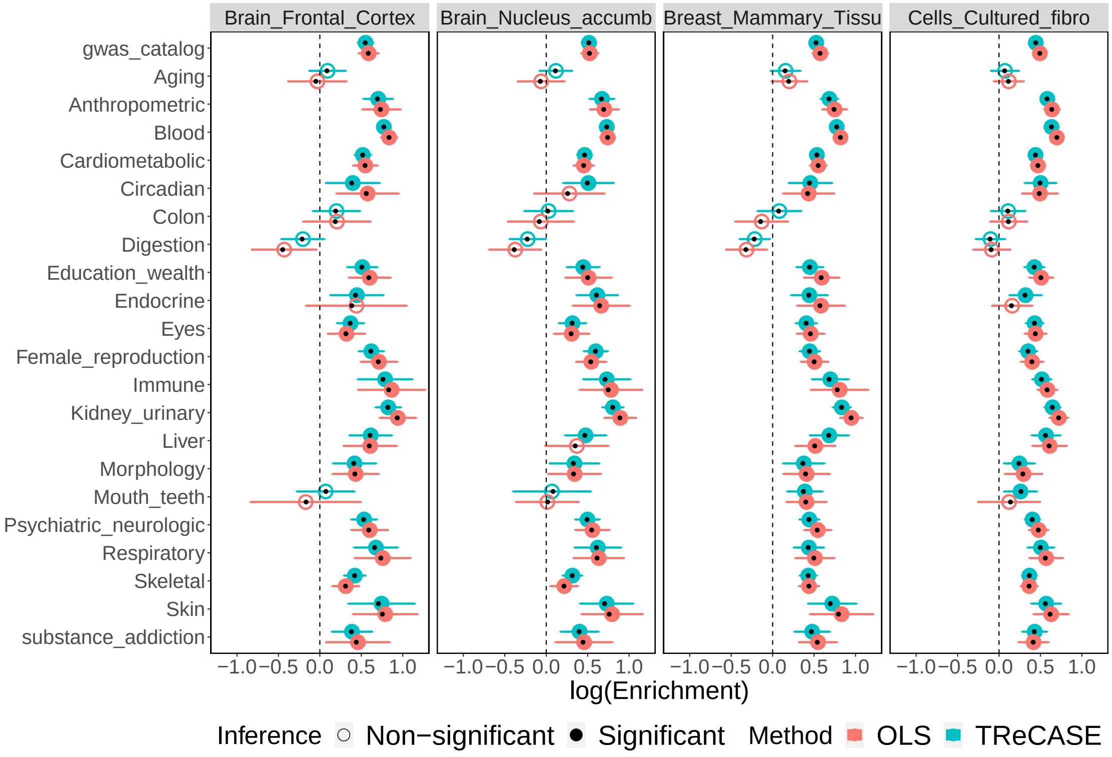

Brain_Frontal_Cortex
Brain_Nucleus_accumb
Breast_Mammary_Tissu
Cells_Cultured_fibro
gwas_catalog
Aging
Anthropometric
Blood
Cardiometabolic
Circadian
Colon
Digestion
Education_wealth
Endocrine
Eyes
Female_reproduction
Immune
Kidney_urinary
Liver
Morphology
Mouth_teeth
Psychiatric_neurologic
Respiratory
Skeletal
Skin
substance_addiction
−1.0 −0.5 0.0 0.5 1.0
log(Enrichment)
Inference
Non−significant
Significant
Method
OLS
TReCASE

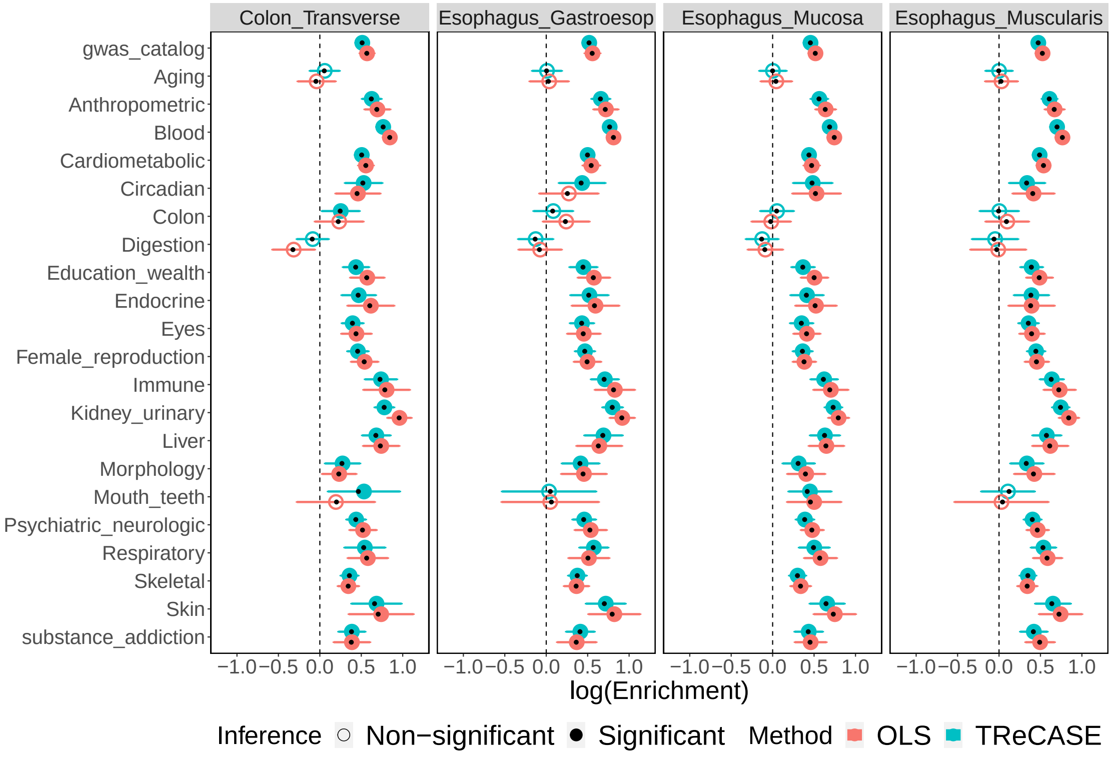
Colon_Transverse
Esophagus_Gastroesop
Esophagus_Mucosa
Esophagus_Muscularis
gwas_catalog
Aging
Anthropometric
Blood
Cardiometabolic
Circadian
Colon
Digestion
Education_wealth
Endocrine
Eyes
Female_reproduction
Immune
Kidney_urinary
Liver
Morphology
Mouth_teeth
Psychiatric_neurologic
Respiratory
Skeletal
Skin
substance_addiction
−1.0 −0.5 0.0 0.5 1.0
log(Enrichment)
Inference
Non−significant
Significant
Method
OLS
TReCASE

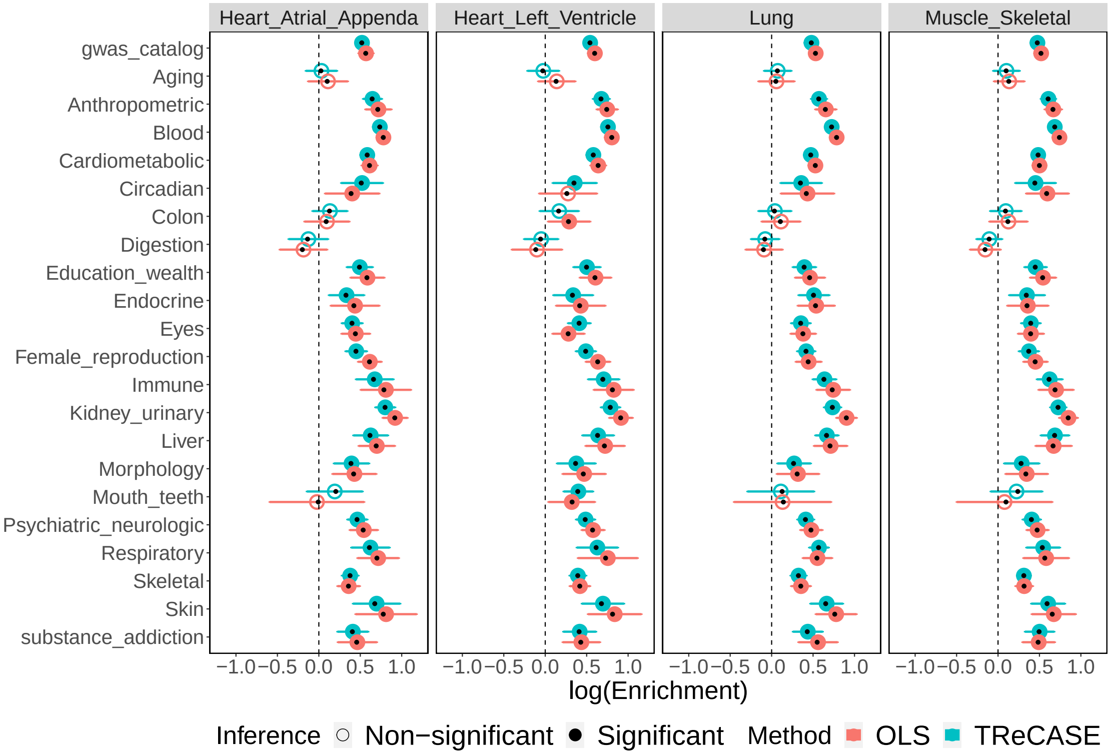
Heart_Atrial_Appenda
Heart_Left_Ventricle
Lung
Muscle_Skeletal
gwas_catalog
Aging
Anthropometric
Blood
Cardiometabolic
Circadian
Colon
Digestion
Education_wealth
Endocrine
Eyes
Female_reproduction
Immune
Kidney_urinary
Liver
Morphology
Mouth_teeth
Psychiatric_neurologic
Respiratory
Skeletal
Skin
substance_addiction
−1.0 −0.5 0.0 0.5 1.0
log(Enrichment)
Inference
Non−significant
Significant
Method
OLS
TReCASE

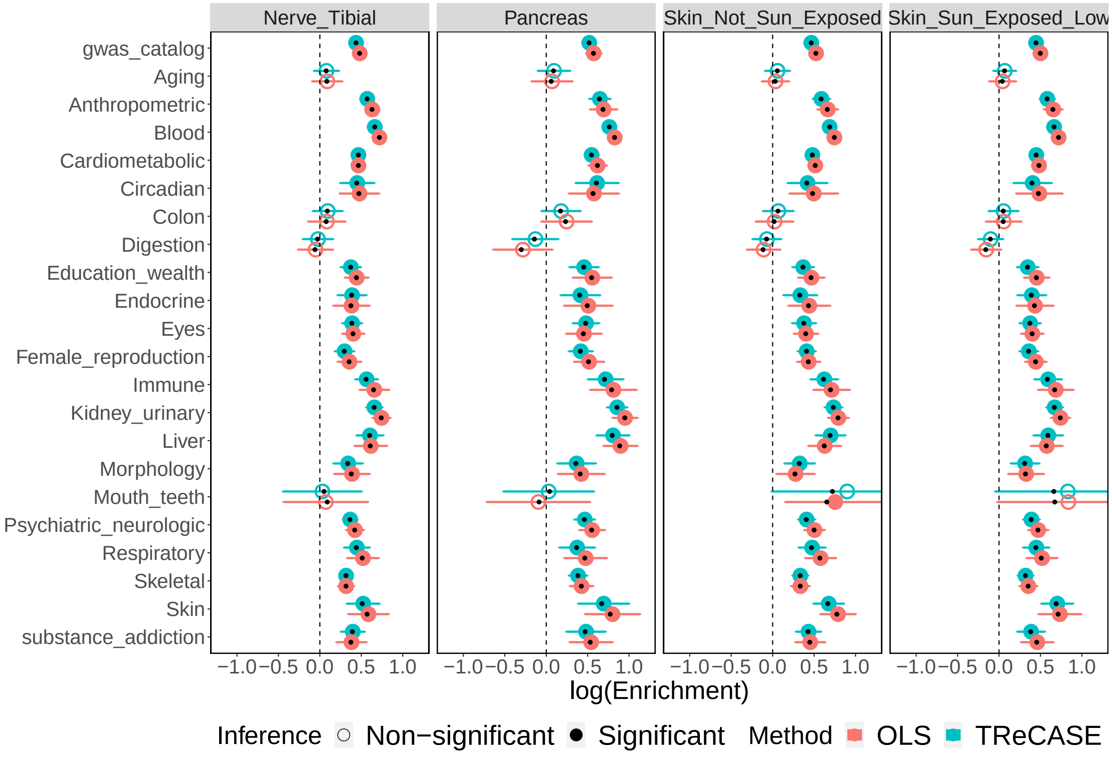

Nerve_Tibial
Pancreas
Skin_Not_Sun_Exposed
Skin_Sun_Exposed_Low
gwas_catalog
Aging
Anthropometric
Blood
Cardiometabolic
Circadian
Colon
Digestion
Education_wealth
Endocrine
Eyes
Female_reproduction
Immune
Kidney_urinary
Liver
Morphology
Mouth_teeth
Psychiatric_neurologic
Respiratory
Skeletal
Skin
substance_addiction
−1.0 −0.5 0.0 0.5 1.0
log(Enrichment)
Inference
Non−significant
Significant
Method
OLS
TReCASE

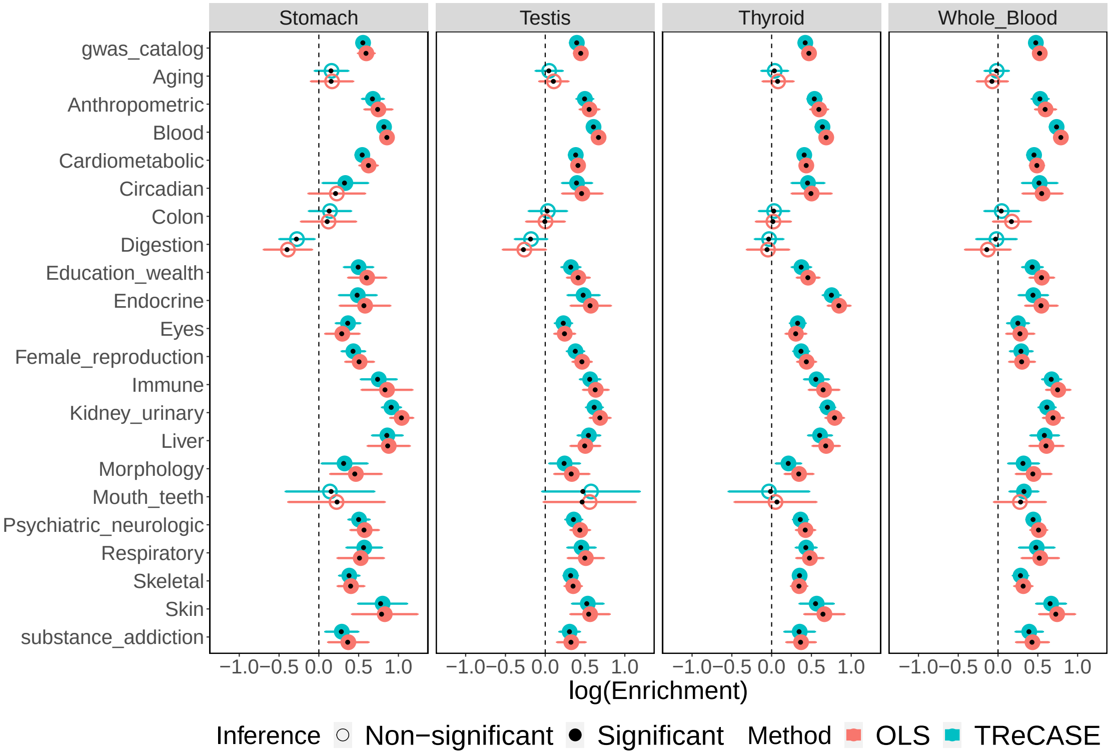

Stomach
Testis
Thyroid
Whole_Blood
gwas_catalog
Aging
Anthropometric
Blood
Cardiometabolic
Circadian
Colon
Digestion
Education_wealth
Endocrine
Eyes
Female_reproduction
Immune
Kidney_urinary
Liver
Morphology
Mouth_teeth
Psychiatric_neurologic
Respiratory
Skeletal
Skin
substance_addiction
−1.0 −0.5 0.0 0.5 1.0
log(Enrichment)
Inference
Non−significant
Significant
Method
OLS
TReCASE

C.4 Supplementary results for dynamic eQTLs.

C.4.1 Cell type proportion estimate from whole blood

In order to estimate cell type proportions, we need to know the relevant cell types of a tissue as well as cell type-specific gene expression. We choose to estimate cell type proportions in whole blood because there is a popular reference of 22 cell types for whole blood, known as LM22, which was compiled by Newman et al. [18]. We first transformed the gene expression data from GTEx (v8, whole blood) to transcript per million (TPM), and then estimated cell type proportions using LM22 reference and CIBERSORTx web interface (`https://cibersortx.stanford.edu/`) [19].

C.4.2 Dynamic eQTLs related with CTCF and TP53

To evaluate the association between CTCF and TP53's expression and seven potential confounders: top 5 PEER factors plus 2 genotype PCs, we fit a linear model with read-depth corrected CTCF or TP53's expression as response variable and the six potential confounders as covariates. These linear models can explain a substantial proportion of the variation of CTCF and TP53's expression (Fig A16a-b). See `https://github.com/Sun-lab/asSeq/blob/master/pipeline_GTEx/v8/cell_type_composition/step3_age_CTCF_TP53.pdf` for more details. After accounting for these six potential confounders, we can still identify some dynamic eQTLs and Fig A16c-d give two examples.

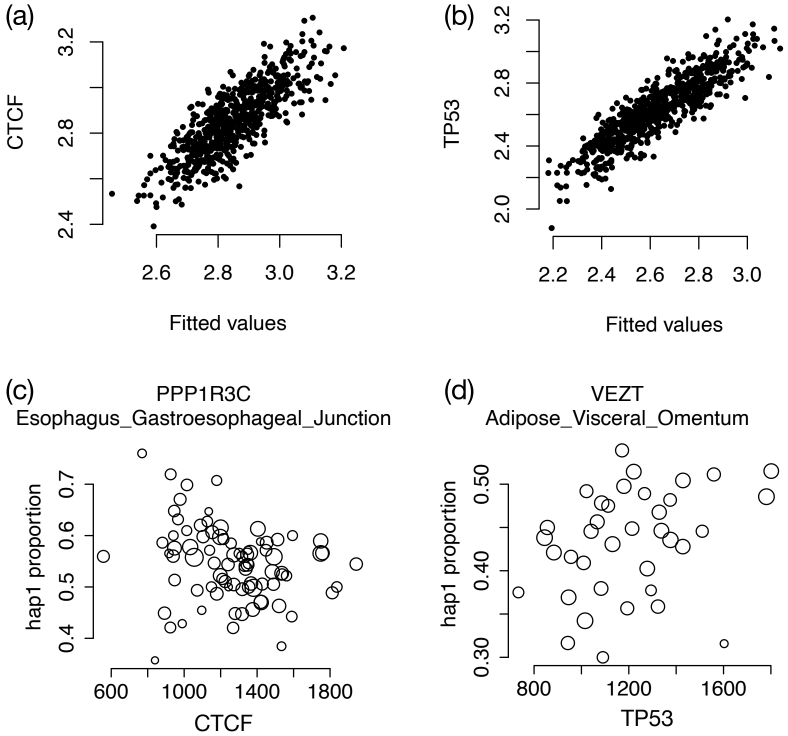

Fig. A16: (a-b) Fitted values from a linear model with seven covariates (top 5 PEER factors plus 2 genotype PCs,) versus observed gene expression for *CTCF* and *TP53*. (c-d) Examples of dynamic eQTLs mediated by the expression of *CTCF* and *TP53*, respectively.

C.5 Evaluation of the binomial distribution assumption for ASReCs across multiple SNPs within one gene and one sample

TReCASE assumes the summation of ASReCs across multiple SNPs within a gene follows a beta-binomial distribution across samples. This assumption implies that the ASReCs across multiple SNPs within a gene and a sample should follow the same binomial distribution. If these SNP-level ASReCs in fact follow a beta-binomial distribution, their summation will not follow a beta-binomial distribution, though it may be approximated by a beta-binomial distribution. Here we evaluate this binomial distribution assumption. It cannot be evaluated for most (gene, sample) pairs because most such cases only have a few heterozygous SNPs with enough coverage. We used RNA-seq data from 30 HapMap trios generated from our earlier study (NCBI BioProject access number: PRJNA385599) [20] to select a set of genes with allele-specific counts distributed across multiple SNPs. This dataset was used since it has higher read depth. We selected those (gene, sample) pairs such that the gene in the sample has at least 6 heterozygous SNPs, with at least 5 overlapping reads per SNP. We ended up with 4,005 (gene, sample) pairs matching these criteria, accounting for less than 1% of all (gene, sample) pairs.

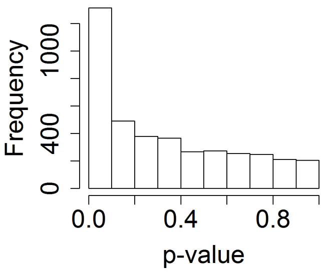

Fig. A17: The distribution of p-values for testing deviation from binomial distribution across multiple SNPs of the same gene and within the same sample

For these 4,005 cases we tested how often the binomial assumption is violated. Deviation from such assumption can be tested using a score statistic developed by [21]. Since we do not have many SNPs, normal approximation of score statistic cannot be applied. Instead, we generated the null distribution of such score statistic using parametric bootstrap. For each gene, we estimated the proportion of reads from one allele, simulated ASReC for each SNP from a binomial distribution with this probability and the observed coverage and recalculated the score statistic. Then a p-value can be calculated as the relative frequency when the observed score statistic is more extreme than the ones from parametric bootstraps. Under the null we expect a uniform distribution of p-values, however we see an excess of

p-values in the category less than 0.1, which transforms to an estimate that the p-value is not uniform for ∼23% of the cases (Fig A17). Note that this 23% is among those selected 1% of the cases with enough heterozygous SNPs covered by RNA-seq reads, and thus only represent a very small proportion of all the data points in real data analysis.

C.6 Simulation Results to compare TReCASE vs. RASQUAL

C.6.1 Simulation results under RASQUAL assumption

Under RASQUAL's assumption, the SNP-level ASReC follows a beta-binomial distribution, such that the similarity of the SNP-level ASReCs are the same for two SNPs within one sample versus two SNPs of two different samples. This is the simulation scenario described in Section B.2.4. It worth noting that this scenario is not supported by real data. As shown in Fig A17, when considering a subset of (gene, sample) pairs with at least 6 heterozygous SNPs and at least 5 overlapping reads per SNP, in 77% of the cases the ASReCs within a sample follow a binomial distribution. We mainly want to use this scenario to demonstrate that RASQUAL does control type I error if its model assumption is correct. In addition, TReCASE model is mis-specified in this scenario, and we demonstrate that TReCASE still has reasonable performance despite its model mis-specification.

As shown in Fig A18, both TReCASE and TReCASE-RL control type I error well except that TReCASE-RL is slightly conservative when negative binomial over-dispersion is 2 and beta-binomial over-dispersion is 0.25. The power of the two methods are similar. TReCASE has slightly higher power when the over-dispersion for beta-binomial distribution is small, and TReCASE-RL has slightly higher power when the over-dispersion for beta-binomial is large.

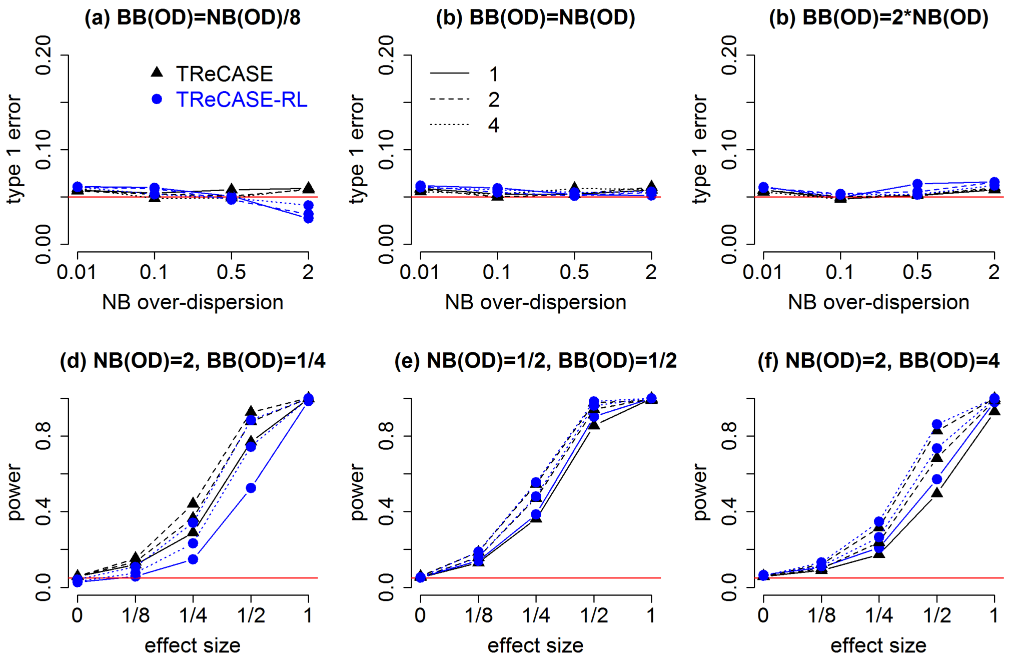

Fig. A18: Evaluating TReCASE and TReCASE-RL using data simulated under RASQUAL assumption such that within sample over-dispersion is the same as between sample over-dispersion. Panels (a)-(c) present type I error and panels (d)-(f) present power. 10,000 genes were simulated for each effect size and over-dispersion profile. The three line types refer to the number of fSNPs per gene. We use sample size 64 in our illustrations.

C.6.2 Simulations results with within sample over-dispersion and additional between sample over-dispersion for ASReCs

This is the simulation setting where both models are mis-specified. Following the simulation described in Section C.3, we simulated ASReC data with within sample over-dispersion and extra between-sample over-dispersion. When simulating two SNPs per gene, TReCASE still manages to control type I error, while the RASQUAL style approach has inflated type I error and the inflation increases for larger beta-binomial over-dispersion (Fig 5(C) in main text). In the case of 4 or 8 SNPs per gene, the results are similar. TReCASE still controls type I error, and RASQUAL style approach shows even higher inflation of type I error (results not shown).

C.6.3 Evaluating models for the data simulated under TReCASE assumption

In previous section we have shown that for the majority of the genes the ASReC do not have within sample over-dispersion. This is a TReCASE style assumption. In this section, we simulated ASReC without within sample over-dispersion, and either combined them into one count or split them across two or four SNPs. Our simulation results show that in this scenario, TReCASE controls type I error reasonably well (Fig A19 (a)-(c)), while RASQUAL can either produce deflated type I error (Fig A19 (a)) or inflated type I error (Fig A19 (b)-(c)).

We also evaluated the effect of double counting by randomly selecting 10% of the reads from each SNP and adding them to a neighboring SNP. Double-counting further inflates type I errors in all three simulation settings (Fig A19 (d)-(f)).

Comparing the results between TReCASE-RL and RASQUAL, we concluded that RASQUAL is has some additional reasons to produces type I error inflation, not explained by equating total read counts and allele-specific counts over-dispersion or treating each SNP count as independent Beta-Binomial as implemented in TReCASE-RL version (Fig A19 (h-j)).

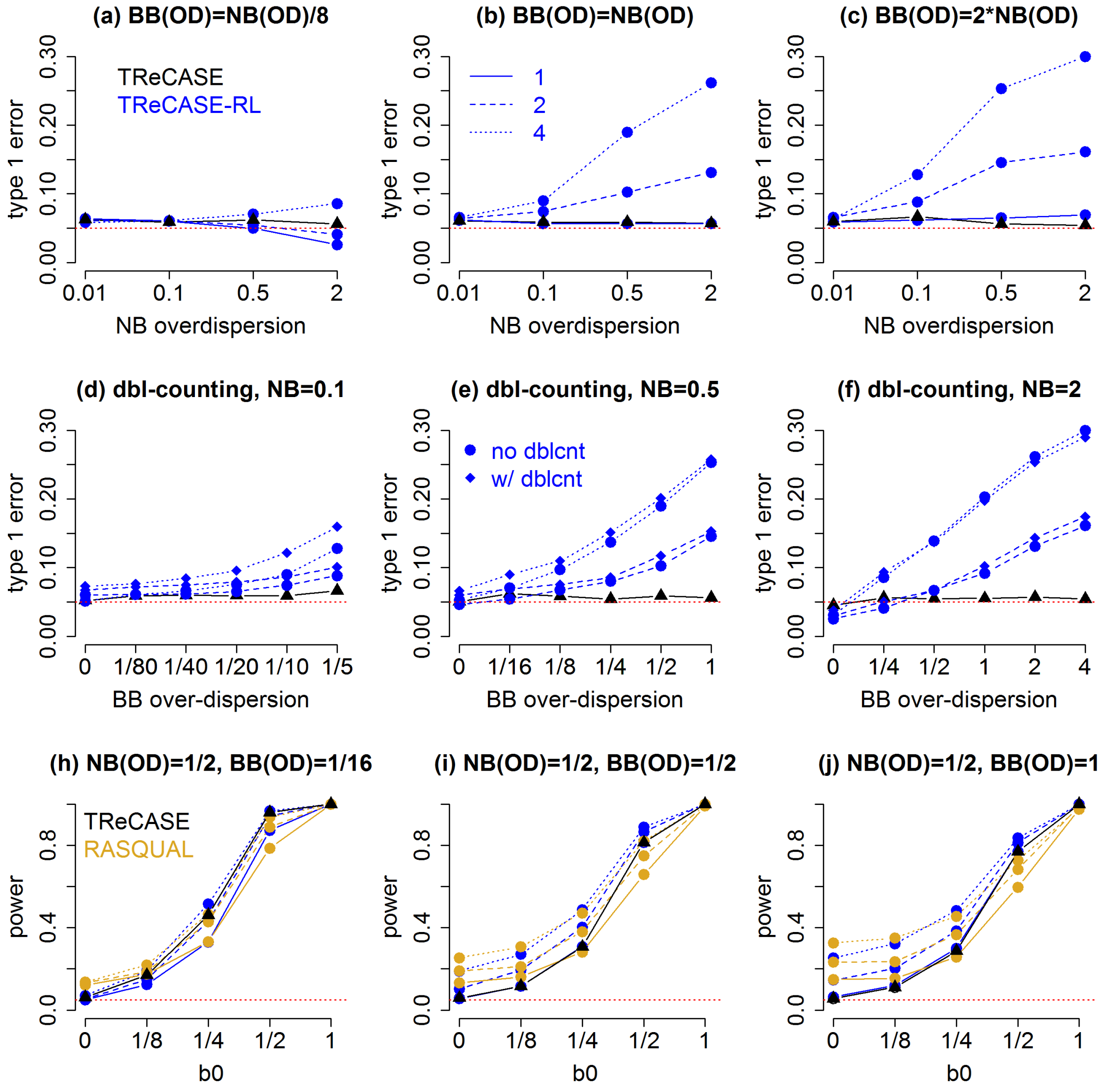

Fig. A19: Evaluating TReCASE, TReCASE-RL, and RASQUAL models for the data simulated using TReCASE style assumptions. a)-(c) Evaluation of Type I error across different values of over-dispersion parameters. (d)-(f) Evaluation of type I error given double counting. (h)-(j) Evaluation of power and type I error across eQTL effect sizes. Results are presented for sample size 64.

C.6.4 Evaluation of the inflation of type I error by RASQUAL

In the previous section, we showed applying RASQUAL or TReCASE-RL on simulated RNA-seq data (using both TReC and ASReC), there is inflated type I error. In this section, we study this issue by ignoring TReC and concentrating on ASReCs without within-sample over-dispersion. We set beta-binomial over-dispersion to 0.1 or 0.5 and generate data under null hypothesis of no eQTL effect (proportion of either allele is set to be 0.5). We simulated data for 1,000 genes. For each gene, we first simulated the data assuming there is only one fSNP. Then we split the ASReCs uniformly to multiple fSNPs, while the total number of allele-specific reads are the same. For example, if there are k reads per SNP for 8 SNPs, then there are $2k$ reads per SNP for 4 SNPs. We applied TReCASE-RL on these data. As shown in previous results, there is no inflation of type I error in one SNP scenario. In two SNP scenario, as expected, both likelihood ratio test-statistics (LRT) and -log(p-value) become larger than one SNP scenario (Fig A20).

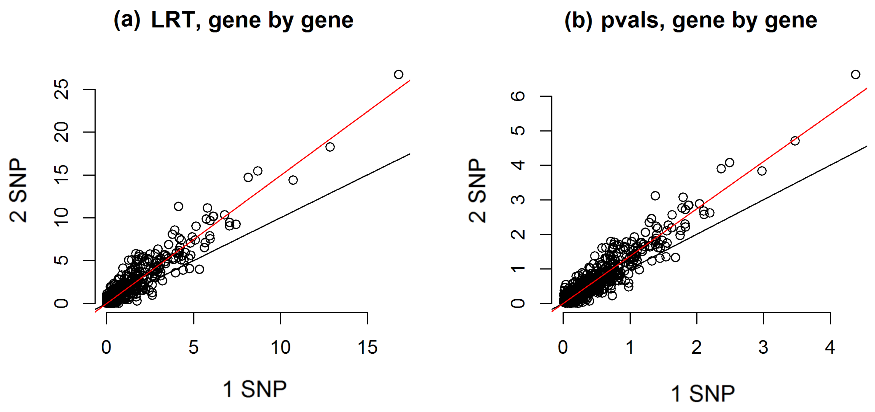

Fig. A20: Illustration of the type I error inflation by TReCASE-RL. The data were simulated under null. Likelihood ratio test-statistics (LRT) and -log10(p-values) are compared for two situations: one fSNP or two fSNPs. Each point corresponds to one of 1000 genes. We observe that for the same gene once reads are split into two SNPs we tend to get more significant results.

To understand the underlying causes of type I error inflation, we examine the estimation of eQTL effect and over-dispersion across simulate replicates. We found that after splitting the allele-specific reads to 2, 4 or 8 fSNPs, the estimation of eQTL effects and over-dispersion are both unbiased (Fig A21). As the number of fSNPs increases, the variation of eQTL effect estimates remain similar while the variation of over-dispersion estimates increases. However, due to the mis-specification of likelihood model by RASQUAL, the mode-based standard deviation (sd:mod in Fig A21) estimates are smaller than observed sds (sd:obs in Fig A21) for both eQTL effects and over-dispersion. The under-estimation of sd for eQTL effects explains the inflation of type I error when we test for eQTL effect. Note that model-based sds do match well with empirical sds when there is only one fSNP. This is expected since there is no model mis-specification when there is only one fSNP.

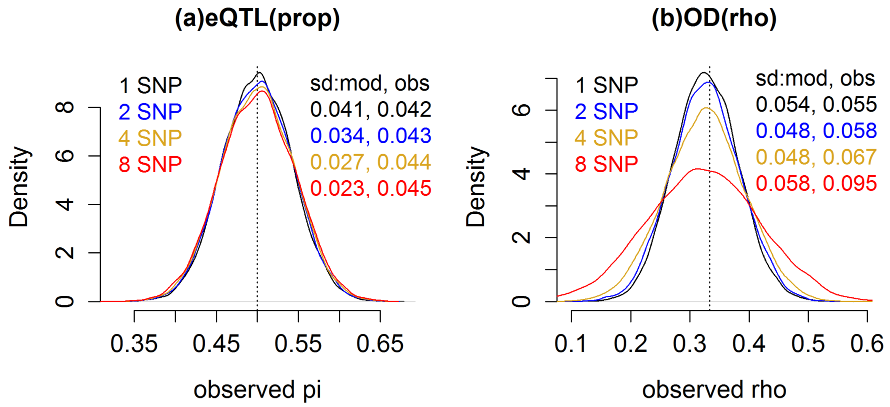

Fig. A21: Distribution of eQTL effect and over-dispersion estimates. (a) Distribution of eQTL effect estimates in terms of π, the proportion of ASReC from one haplotype. (b) Distribution of over-dispersion estimates in terms of ρ, which is the rescaling of over-dispersion parameter θ to $[0, 1)$ range by $\rho = \theta/(1 + \theta)$. In the upper-right corner of each figure, we also list the model-based standard deviation estimate (sd:mod) using Fisher's information matrix [15] and empirical standard deviation estimate (sd:obs) across simulation replicates. The data were simulated under null of no genetic effect, and ASReCs were split into 1, 2, 4, and 8 SNPs. Simulation was done for sample size 64 and on average 10 allele-specific reads per sample.

We derive sd using the Fisher's information matrix derived by [15], and to be consistent with their work, we quantify over-dispersion by $\rho = \theta/(1+\theta)$, where θ was the over-dispersion parameter defined in Equation (1). To make the notation clear, we use legend OD(theta) or OD(rho) in the plots to indicate over-dispersion quantified by θ and ρ, respectively.

Next, we examine how the model-based sd estimates vary with respect to the number of allele-specific reads. As expected the sd estimates of either eQTL effect and over-dispersion decreases as ASReC increases (Fig A22). For eQTL effect size, we have observed in Fig A21 that the true sds are similar when the number of fSNPs is 1, 2, 4, or 8. Since the sd estimate of 1 SNP scenario is unbiased, the difference of the sd estimates with 1 SNP versus 2, 4, or 8 SNPs reflects bias of sd estimates due to model mis-specification (Fig A22 (a) and (c)). The standard deviation of over-dispersion parameter is of less interest in this study. Though we can see that when the number of ASReC is relatively large, the sds of over-dispersion tend to be under-estimated (Fig A22 (b) and (d)).

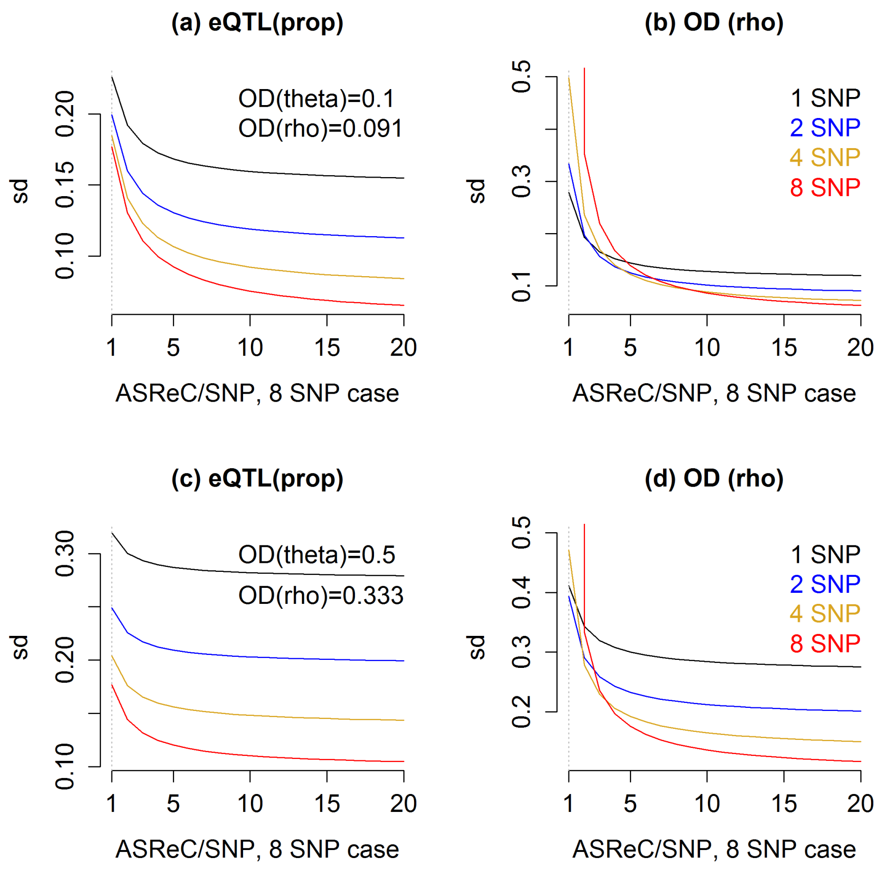

Fig. A22: Model-based sd estimates by Fisher's information matrix under null. The sd estimates are evaluated for 1, 2, 4, and 8 fSNPs per gene. X-axis is the number of allele-specific reads for each of 8 fSNPs. For example, $x = 5$ means there are 5 reads for each of the 8 SNPs, or 10 reads for each of the 4 SNPs, or 20 reads for each of the 2 SNPs, or 40 reads for one SNP. Panels (a)-(b) present the simulation results for $\phi = 0.1$ and panels (c)-(d) present the simulation results for $\phi = 0.5$

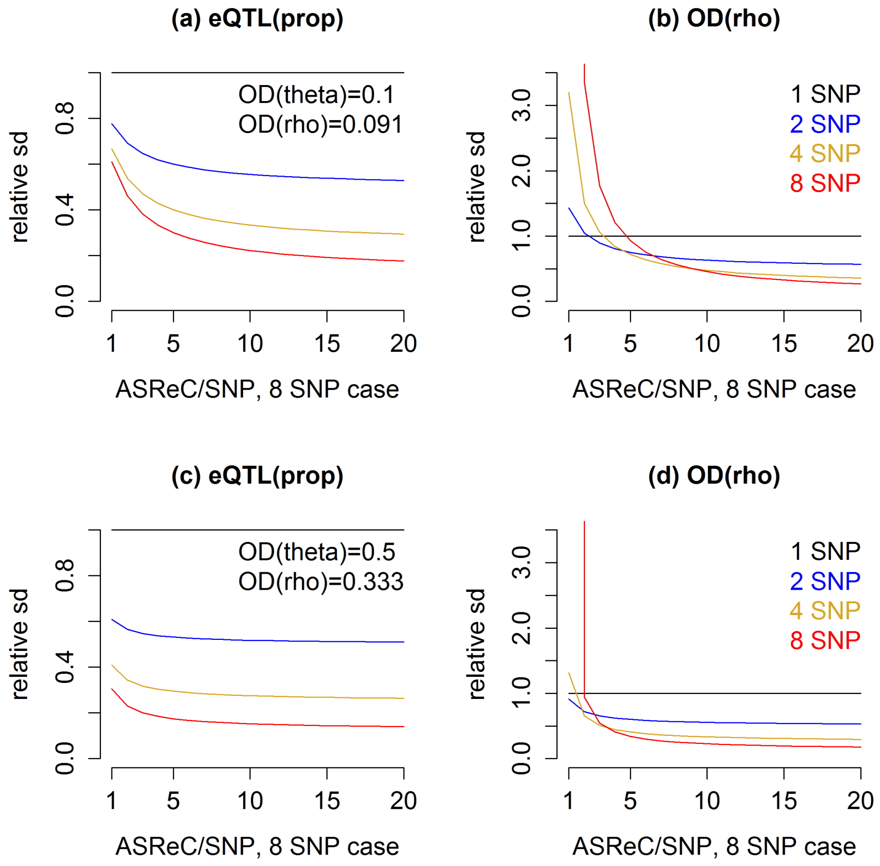

Fig. A23: Model-based relative sd estimates under null.

The same as Fig A22, except that the y-axis is the relative sd estimates with respect to the sd estimate for 1 fSNP scenario.

C.6.5 Compare TReCASE versus RASQUAL when there are genotyping errors

Finally, we evaluate the results of TReCASE and RASQUAL when there are genotyping errors using simulated data. We simulated ASReC data without within sample over-dispersion (Section B.2.2), and then introduced genotyping errors as described in Section B.2.5, where we flipped certain fraction of genotypes from homozygous to heterozygous and from heterozygous to homozygous. When truly heterozygous SNPs are listed as homozygous, they will be discarded by TReCASE but used by RASQUAL, and the latter has a mechanism of correcting the genotype status if it encounters a conflicting SNP. If truly homozygous SNPs are listed as heterozygous, TReCASE will take them at face value while RASQUAL again will try to correct them. Because RASQUAL needs to use multiple SNPs to correct a wrong one, we only consider a scenario when splitting counts to 8 SNPs. Since most genes have less than 8 heterozygous SNPs, these simulation results represent an uncommon situation that favor the genotyping error correction mechanism of RASQUAL.

We consider the simulation setting when the over-dispersion parameters of negative binomial and beta-binomial are the same to match with the assumption made by RASQUAL. We illustrate the type I errors and powers for this simulation setup for a few values of over-dispersion parameters and several sample sizes (Fig A24). TReCASE controls type I error in all simulation setups. In contrast, RASQUAL controls type I error only if sample size is small and over-dispersion is small (Fig A24 (d)-(f)). The power of TReCASE remains similar as the proportion of genotyping errors increases, with some slight reduction of power when over-dispersion is large and eQTL effect size is large. Larger fraction of genotyping errors also reduces the power of RASQUAL in a magnitude slightly larger than for TReCASE (Fig A24 (a)-(c)).

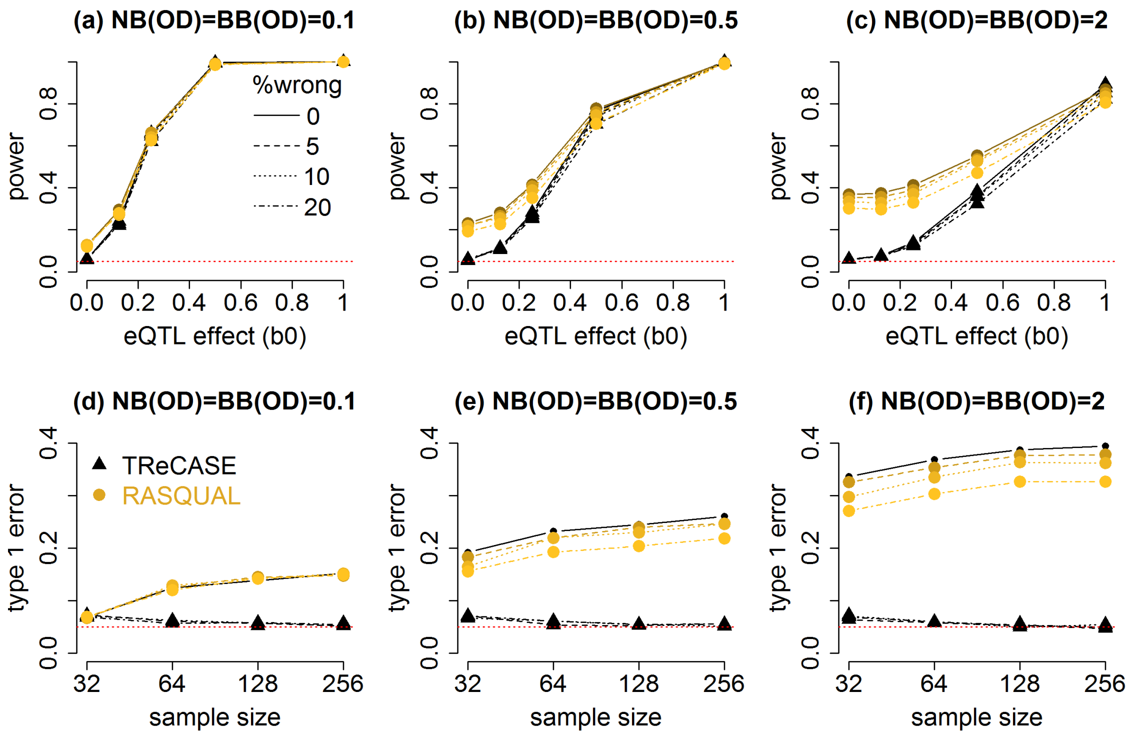

Fig. A24: Type I errors and powers for a fitting TReCASE and RASQUAL with 0, 5%, 10%, or 20% of all the fSNPs being randomly flipped between homozygous and heterozygous. Sample size 64 was used.

C.7 Compare MatrixEQTL, TReCASE, and RASQUAL using Geuvadis dataset

C.7.1 Computational time

We performed eQTL mapping for all the genes for which there were at least 5 samples with at least 5 allele-specific reads. Since RASQUAL is very time consuming, we fit each gene in parallel. For timing comparability, we did the same for TReCASE. We limited total computational time per gene to be a week, and RASQUAL failed to finish within a week for 9 genes. We summarize the results for the remaining 14,427 genes. The average number of potential eQTL SNPs per gene was 2,000. The computational time of both TReCASE and RSQUAL increases nearly linearly with sample sizes, and TReCASE is more than 10 times faster than RASQUAL (Fig A25(a)). For the full dataset with sample size 280, if all the computation were done without any parallel computing, RASQUAL took 610 days and TReCASE took 53 days (Table A6).

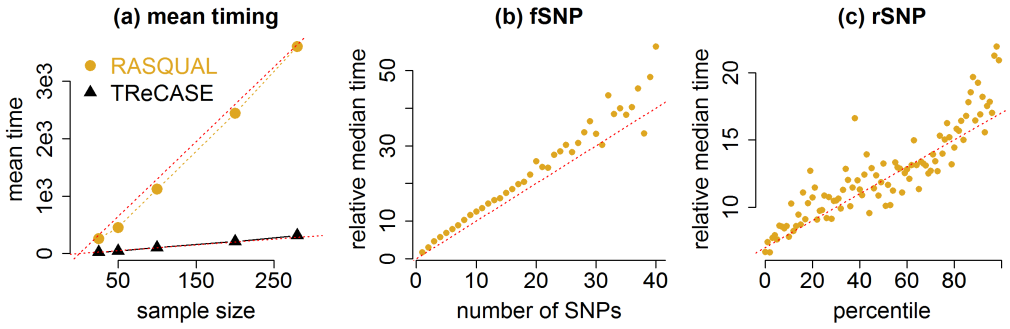

Fig. A25: TReCASE vs RASQUAL timing for eQTL mapping. (a) The average computational time (seconds) for eQTL mapping per gene versus sample size. The dotted lines are $y = x$ and $y = 13x$. (b-c) The median of relative time for eQTL mapping per gene using RASQUAL versus TReCASE versus the number of feature SNPs (fSNPs) (with a reference line $y = x$) or the number of eQTL SNPs (rSNPs) (with a reference line $y = 7 + 0.1x$).

We also include the computational time for permutation p-value estimation by our method geoP, which uses a logistic regression to estimate the relation between nominal p-value and permutation p-value. Because the estimation of permutation

p-value uses computationally very efficient linear regression implemented in MatrixEQTL [22], its computational time is comparable to one run of TReCASE. For 14,500 genes with 25, 50, 100, and 200 grid points per gene, it takes approximately 19, 23, 28, and 42 days, respectively (Table A6). Although it still takes substantial amount of time, it can be easily paralleled gene by gene.

Table A6: Comparison of computational time in days. 'one run': time for one round of eQTL mapping. 'est.perm.pval': time for estimation of permutation p-values using 100 grid points, or the range of computational time shown in the parenthesis using 25 to 200 grid points. 'total time': the total time needed for eQTL mapping and estimation of permutation p-values. The last row of the table shows the computational time of a modified version of TReCASE [23] where a score test is developed that improves computational efficiency. The TReCASE(score) method does not separate the initial run and permutation p-value calculation and thus we record the total time using 5,000 permutations.

method	one run	est. perm.pval	total time
RASQUAL	610	28 (19-42)	638 (629-652)
TReCASE	53	28 (19-42)	81 (72-95)
TReCASE (score)	-		750 (750)

We have developed a modified version of TReCASE to perform testing using score test [23]. This TReCASE (score) method is computationally more efficient to perform permutations since some elements of the score test statistic can be calculated only once and used for many permutations. For 280 samples in the Geuvadis dataset, TReCASE (score) took 750 days for 5,000 permutations (Table A6), and thus it is doable within a week using 100+ computing jobs. However, one limitation of score test is that the p-values become less stable when sample size is relatively small, such as n=50 or 100. While the permutation p-values are still accurate, one should be cautious when using the nominal p-values with small sample sizes.

The relative computational time of RASQUAL versus TReCASE increases with respect to the number of fSNPs, i.e., the feature SNPs on which ASE is measured (Fig A25(b)) and the number of rSNPs , i.e., eQTL SNPs (Fig A25(c)). When geno-

type data were obtained by whole genome sequencing (e.g., GTEx dataset), there are about 10 times of fSNPs per gene (Fig A26) than the genotype data obtained from imputation (e.g., Geuvadis dataset). As a consequence, RASQUAL can be 100 times slower than TReCASE. For example, we compared the computational time of TReCASE and RASQUAL using GTEx v7 whole blood data, using a smaller local-eQTL window of 100kb flanking regions + gene body. Using a single CPU, TReCASE takes 14 days while RASQUAL takes 1,663 days. For this reason, we do not evaluate RASQUAL using the GTEx v8 data.

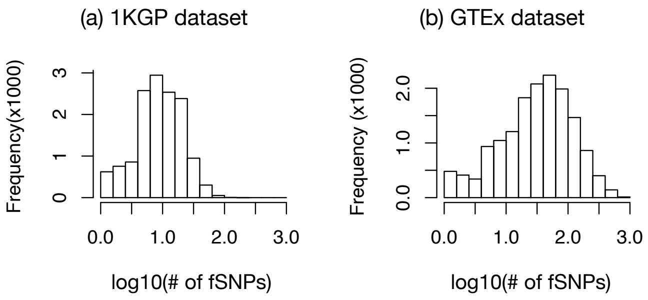

Fig. A26: Number of fSNPs per gene. The distribution of the number of fSNPs per gene for (a) Geuvadis (1KGP) dataset or (b) GTEx dataset (whole blood, v7), respectively.

C.7.2 Choose a permutation p-value cutoff to control FDR

Given the permutation p-value for each gene, we can choose a permutation p-value cutoff to control FDR by evaluating q-value. We estimated the fraction of genes that follow the null distribution (denoted by π_0) by doubling the fraction of the genes with permutation p-values above 0.5, and then estimate FDR (or an approximation of q-value) for permutation p-value α by $\pi_0 \alpha N / D_\alpha$, where N is the total number of genes and D_α is the number of genes with permutation p-value $\leq \alpha$. Since a larger proportion of genes has significant eQTLs, π_0 can be small and thus for some FDR cutoffs, the corresponding permutation p-value can be even larger than the FDR (Table A7). For example, to control FDR at 0.05, the p-value cutoffs are larger than 0.05. Here we use FDR cutoff 0.01 to choose permutation p-value cutoffs for the three methods.

Table A7: Permutation p-value cutoffs for different FDR cutoffs, using Geuvadis dataset with sample size 280.

FDR	TReCASE	RASQUAL	MatrixEQTL
0.001	0.001	0.0009	0.0005
0.01	0.014	0.011	0.009
0.05	0.092	0.071	0.074
0.1	0.211	0.161	0.189
0.2	0.474	0.362	0.477
0.25	0.616	0.470	0.631

C.7.3 Compare TReCASE vs. MatrixEQTL

As illustrated in Fig 2 of main text, TReCASE has much higher power than MatrixEQTL when sample size is relatively small (e.g., $n \leq 140$). However, such results do not answer a related but different question: how many of the eQTLs overlap and how do such overlaps change with respect to sample size. We first show that TReCASE vs. MatrixEQTL tend to identify the same set of eQTLs when sample size is large enough because the correlations of their p-values (in log scale) increases with sample size (Table A8).

Table A8: Correlations of TReC or TReCASE p-values vs MatrixEQTL p-values on -log10 scale

Sample size (n)	TReC	TReCASE
35	0.72	0.67
70	0.89	0.77
140	0.95	0.85
280	0.97	0.87

Next, we compare the overlaps of the eQTL findings across sample sizes using q-value 0.01 as cutoff. Because we have more confidence on the eQTLs identified with larger sample sizes, we made three comparisons where each time, we compared the results of all the methods at all sample sizes versus the eQTL findings by one method using the maximum sample size of 280. TReCASE can identify most eGenes reported by MatrixEQTL (i.e., 95% when n=280) while MatrixEQTL still misses 42% of the eGenes identified by TReCASE when n=280 (Tables A9 and A11). Since both MatrixEQTL and TReC use total expression but not ASE, as expected there is higher consistency when comparing MatrixEQTL vs. TReC than comparing MatrixEQTL vs. TReCASE (Table A10). It is also clear that sample size 35 is too small for reliable eQTL mapping. MatrixEQTL report no findings when n=35 and 19% of TReCASE eQTL findings cannot be found when sample size is 280.

Table A9: Comparison to the eGenes identified by TReCASE when sample size (n) is 280. The columns of "Recovered eGenes" are the proportion of TReCASE eGenes (when n=280) recovered by each method at each sample size. The columns of "eGenes missed by TReCASE" are the proportion of eGenes identified by each method (at each sample size) but missed by TReCASE when n=280.

	Recovered eGenes			eGenes missed by TReCASE		
n	MatrixEQTL	TReC	TReCASE	MatrixEQTL	TReC	TReCASE
35	0	0.02	0.05	-	0.26	0.19
70	0.03	0.06	0.14	0.14	0.09	0.08
140	0.19	0.24	0.45	0.06	0.05	0.06
280	0.58	0.67	1	0.05	0	0

Table A10: Comparison to the eGenes identified by TReC when n=280.

	Recovered eGenes			eGenes missed by TReC		
n	MatrixEQTL	TReC	TReCASE	MatrixEQTL	TReC	TReCASE
35	0	0.03	0.07	-	0.36	0.28
70	0.04	0.08	0.18	0.15	0.11	0.17
140	0.27	0.34	0.54	0.08	0.08	0.23
280	0.81	1	1	0.09	0	0.33

Table A11: Comparison to the eGenes identified by MatrixEQTL when n=280.

	Recovered eGenes			eGenes missed by MatrixEQTL		
samples	MatrixEQTL	TReC	TReCASE	MatrixEQTL	TReC	TReCASE
35	0	0.03	0.07	-	0.37	0.28
70	0.05	0.09	0.2	0.09	0.13	0.2
140	0.31	0.37	0.57	0.07	0.12	0.28
280	1	0.91	0.95	0	0.19	0.42

C.7.4 Genomic locations of eQTLs

For each method, we selected the eGenes with q-value ≤ 0.01 and for each eGene, we selected the SNP with the most significant p-value. Then we summarized the location of those eQTLs with respect to the corresponding genes. We observe enrichment around transcription starting site regardless of the strand of each gene (Fig A27). There is also a relatively weaker enrichment around transcription ending site (TES). Note that few eQTLs are located more than 10kb away from the corresponding gene. We also tried several cutoffs and noticed that when we select eQTLs with smaller p-value and thus reducing fraction of false positive discoveries, the fraction of eQTLs farther away from gene goes down.

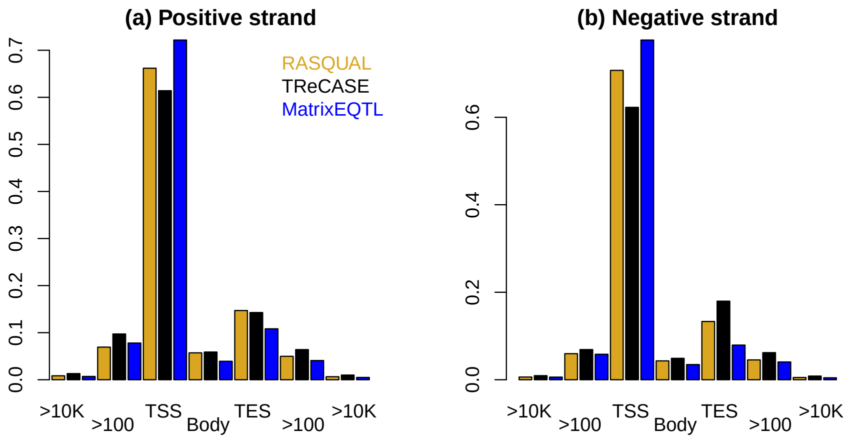

Fig. A27: The distribution of the most significant eQTL for each eGene (q-value $\leq$ 0.01). The region "TSS" or "TES" are defined as the region of 200bp (i.e., 100 bp on either side) around transcription starting site (TSS) or transcription ending site (TSS), respectively. The regions labeled ">100" are between 100 and 10kb away from TSS or TES.

C.7.5 Compare the results of RASQUAL vs. TReCASE

RASQUAL estimates one over-dispersion that is shared by its negative binomial and beta-binomial components and TReCASE estimates the over-dispersion parameters for these two components separately. We observe a quite clear pattern that the

over-dispersion estimate from RASQUAL is very similar to the over-dispersion estimate of TReCASE negative binomial (TReC) component, but they are often much larger than the over-dispersion estimate of TReCASE beta binomial (ASE) component (Fig A28).

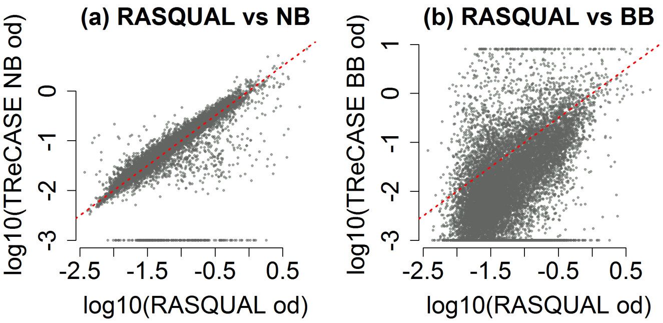

Fig. A28: Comparing estimates of over-dispersion by RASQUAL or TReCASE. (a) TReCASE negative-binomial over-dispersion estimates and (b) TReCASE beta-binomial over-dispersion estimates. We trimmed log10(over-dispersion values) from TReCASE output to $[-3, 1]$ range.

We classified the fitted genes by their significance according to each of two methods (Table A12). Although the results of the two methods are consistent for the majority of the genes, there is some notable discrepancy for a subset of genes.

Table A12: Number of genes passing corresponding cutoff of q-values applied on permuted p-values.

TReCASE	$[0.01, 1]$	$[10^{-3}, 0.01)$	$[10^{-4}, 10^{-3})$	$[10^{-5}, 10^{-4})$	$<$ 1e-5	total
RASQUAL						
$[0.01, 1]$	5463	1544	520	163	140	7830
$[10^{-3}, 0.01)$	363	441	366	174	192	1536
$[10^{-4}, 10^{-5})$	148	161	215	161	251	936
$[10^{-5}, 10^{-4})$	78	100	112	91	242	623
$<$ 1e-5	323	217	243	270	2449	3502
total	6375	2463	1456	859	3274	14427

To identify the potential sources for discrepancies, we conducted the following analyses. For each gene, we took the smallest p-value across multiple rSNPs by TReCASE and RASQUAL, truncated them at 10^{-15}, and refer them as TReCASE p-value and RASQUAL p-value, respectively. We sought to explore the discrepancies of TReCASE and RASQUAL p-values by a linear regression with

$$y = \log_{10}(\text{TReCASE p-value}) - \log_{10}(\text{RASQUAL p-value})$$

as the response variable and 16 covariates (Tables A13-A14):

- alternative allele frequency
- $\log_{10}$ p-value of χ^2 test for Hardy Weinberg equilibrium
- estimated mapping error (Delta), which is an output of RASQUAL
- reference allele bias (Phi Bias), which is an output of RASQUAL
- the number of feature SNPs per gene, centered to median.
- the number of rSNPs per gene, on log scale.
- over-dispersion from total read counts (OD_{NB}) estimated by TReCASE, in log scale and centered.
- over-dispersion from allele-specific counts (OD_{BB}) estimated by TReCASE, in log scale and centered.
- the total allele-specific counts by RASQUAL, in log scale and centered.
- the total allele-specific counts by TReCASE, in log scale and centered.
- interaction of the previous two counts. Two methods count allele-specific reads differently. TReCASE count them at gene level while RASQUAL count them SNP by SNP. Therefore, if one read overlaps with two fSNPs, it will be counted twice. The potential degree of over-counting is illustrated at Fig A30.

- interactions of three covariates: RASQUAL allele-specific counts, the number of fSNPs, and beta binomial over-dispersion. Based on our simulations and study of information matrix we believe that p-value inflation of RASQUAL is caused by its model of ASReC. These three covariates are all important for the ASReC model.

- median p-value of RASQUAL across all rSNPs of a gene, using permuted genotype. This quantity measures the magnitude of RASQUAL type I error for this gene.

All covariates were normalized to have standard deviation of 1.

Table A13: Type 1 (sequential) and Type 3 (added last) ANOVAs for linear regression analysis of $\log_{10}$(TReCASE p-value) - $\log_{10}$(RASQUAL p-value). The direction (Dir.) indicates whether RASQUAL (R) or TReCASE(T) has smaller p-value.

Parameter	Dir.	Type 1 R^2	P-val	Type 3 R^2	P-val	Marg. R^2
OD_{BB}	R	6.7	6e-244	8.2	3e-294	6.7
ASReC_R	R	2.7	8e-101	1	2e-39	0.6
n-fSNP	R	1.9	1e-71	1.6	4e-63	2.7
ASReC_R :n-fSNP	R	0.5	2e-19	0.7	4e-26	0
ASReC_R :OD_{BB}	R	5	1e-185	3.2	1e-118	1.3
n-fSNP:OD_{BB}	R	0.8	6e-31	1	1e-37	0.4
ASReC_R :n-fSNP:OD_{BB}	R	0.2	4e-8	0.2	2e-10	0.1
n-rSNP	T	0	0.73	0	0.52	1
OD_{NB}	T	0	0.009	0	0.004	0.8
ASReC_T	T	0.4	1e-15	0.4	3e-16	0.1
ASReC_R :ASReC_T	R	0.3	6e-13	0.2	1e-10	0
AF	T	0	0.004	0.1	0.002	0
HWE χ^2	T	0.2	5e-9	0.2	1e-8	0.3
Mapping error	T	0	0.09	0	0.24	1.1
Ref. Allel Bias	R	0.3	2e-13	0.3	2e-13	2.1
Med(perm-p)	T	0	0.97	0	0.97	0.4

This linear model explains 15% of the variance of y. More variance of y can be explained by this model if we only consider a subset of genes with more discrepant p-values. For example, for a subset of genes passing a cutoff of $|y| \geq 5$ or $|y| \geq 10$,

Table A14: Linear regression of $y = \log_{10}$(TReCASE p-value) - $\log_{10}$(RASQUAL p-value) versus a set of potential factors. OD_{BB} and OD_{NB} indicate $\log_{10}$ over-dispersion for beta-binomial and negative binomial, respectively. n-fSNP and n-rSNP indicate the number of feature SNPs and regulatory SNPs, respectively. Subscript $_R$ and $_T$ indicate RASQUAL and TReCASE, respectively.

Parameter	Est	SE	P-val	Marg.Est	Marg P-val
intercept	0.18	0.14	0.2	0.31	1.6e-43
OD_{BB}	1.04	0.03	3e-294	0.68	3e-213
$ASReC_R$	0.81	0.06	2e-39	0.20	4e-19
n-fSNP	0.44	0.03	4e-63	0.43	4e-84
$ASReC_R$:n-fSNP	0.24	0.023	4e-26	0.013	0.51
$ASReC_R$:OD_{BB}	0.52	0.022	1e-118	0.26	7e-42
n-fSNP:OD_{BB}	0.29	0.022	1e-37	0.13	2e-12
$ASReC_R$:n-fSNP:OD_{BB}	0.11	0.017	2e-10	-0.04	0.0027
n-rSNP	-0.014	0.022	0.52	0.26	6e-32
OD_{NB}	-0.07	0.024	0.004	0.24	1e-26
$ASReC_T$	-0.46	0.056	3e-16	0.084	2e-4
$ASReC_R$:$ASReC_T$	0.12	0.019	1e-10	0.037	0.048
AF	-0.061	0.02	0.002	-0.044	0.046
HWE χ^2	-0.12	0.02	1e-8	-0.16	3e-12
Mapping Error	-0.028	0.024	0.24	0.28	2e-36
Ref. Allel Bias	0.19	0.026	2e-13	0.38	6e-65
Med(perm p)	-0.001	0.034	0.97	-0.16	5e-13

this linear model explains 42% or 57% of the variance of y, respectively. We note that the more discrepant set of genes we select, the higher fraction of genes with smaller p-values by RASQUAL (Fig A29 (a)), suggesting stronger discrepancies are more likely due to the inflation of Type I error by RASQUAL.

We observe that three factors, beta-binomial over-dispersion (OD_{BB}), the number of fSNPs (n-fSNP), and RASQUAL style ASReC ($ASReC_R$), along with interactions of these terms have strongest associations with the discrepancy of the two methods (Table A14). Larger values of these three factors are all associated with smaller RASQUAL p-values. This is consistent with our findings that the beta-binomial component of RASQUAL treats multiple fSNPs within a sample as independently

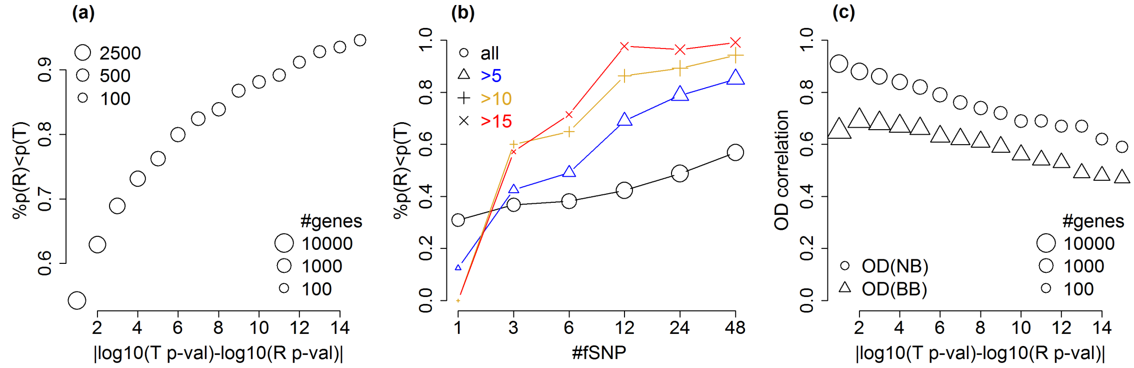

Fig. A29: Comparison of TReCASE and RASQUAL results using Geuvadis dataset. We use “T p-val” and “R p-val” as abbreviations of “TReCASE p-value” and “RASQUAL p-value”, respectively. “%p(R) $<$ p(T)” denotes the proportion of genes with RASQUAL p-value smaller than TReCASE p-value. (a) Genes with more discrepant p-values tend to smaller RASQUAL p-values. (b) The genes with larger number of fSNPs also tend to have smaller RASQUAL p-values. Different point symbols indicate the genes with the absolute value of the difference between $\log_{10}$(TReCASE p-value) and $\log_{10}$(RASQUAL p-value) is larger than certain threshold. (c) When there are larger discrepancies of p-values, the over-dispersion estimates by RASQUAL are less similar to either negative binomial (NB) or beta-binomial (BB) over-dispersion estimates by TReCASE.

distributed, which causes larger inflation of type I error. In addition, more discrepant genes also tend to have weaker correlations of over-dispersion estimates between TReCASE and RASQUAL (Fig A29 (c)).

Among other covariates, the following relations are notable. Significant associations with reference allele bias and Hardy-Weinberg disequilibrium suggest the advantage of RASQUAL to model these factors. Smaller TReCASE p-values are associated with the case when we observed relatively higher TReCASE style allele-specific counts (ASReC_T), which suggests that the ASReC by RASQUAL and TReCASE are different, most likely due to double counting by RASQUAL (Fig A30).

Median RASQUAL p-value using permuted data (Med(perm p)) is very signifi-

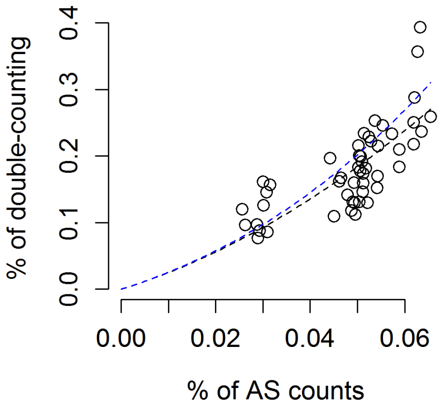

Fig. A30: Estimating double-count in real data

We again used 30 samples from PRJNA385599. For each sample we counted number of allele-specific reads using our TReCASE procedure and produced fraction with respect to total number of reads (x) and will plot on x scale. In addition, for the reads overlapping several heterozygous SNPs we counted such read several time - once for each SNP (define this number as z) Z is inflated with overcounting. We quantify this excess of counts by defining $y = z/x - 1$ and plotting them on the y axis. 10 of the samples in this dataset were measured with both 150bp reads and shorter 75bp reads. They are plotted separately with 10 points around 3% allele-specific counts representing summary for shorter reads.

cant in marginal model, but becomes much less significant in the joint model. This is expected because inflation of type I error is also associated with other factors in the join model (see Section C.6.4 for more details). In both cases, smaller RASQUAL p-value using permuted data are associated with smaller RASQUAL p-values.

We further examine the discrepancy of significant findings by RASQUAL and TReCASE with respect to the number of fSNPs. We classified the genes to be significant or not at several FDR cutoffs and plotted them versus the number of fSNPs. The fraction of significant findings of both methods generally grows with respect to the number of fSNPs (Fig A31(a) and (d)), which is expected since the number of allele-specific reads would also be higher with more fSNPs. However, this fraction

grows quicker for RASQUAL than TReCASE, and it increases regardless the significance level of TReCASE (Fig A31(b) and (c)). This suggests that the association between the number of fSNPs and RASQUAL p-values may not depend on the actual strength of eQTL signals and thus implies inflated type I error. In contrast, conditioning on being significant or insignificant using RASQUAL method we see much weaker association between the number of fSNPs and the fraction of significant TReCASE findings (Fig A31(e) and (f)). In fact, given significant RASQUAL results, the number of significant TReCASE findings has slight decrease as number of fSNPs increases (Fig A31(e)). This is likely because RASQUAL tends to find higher fraction of false positive when the number of fSNPs is large.

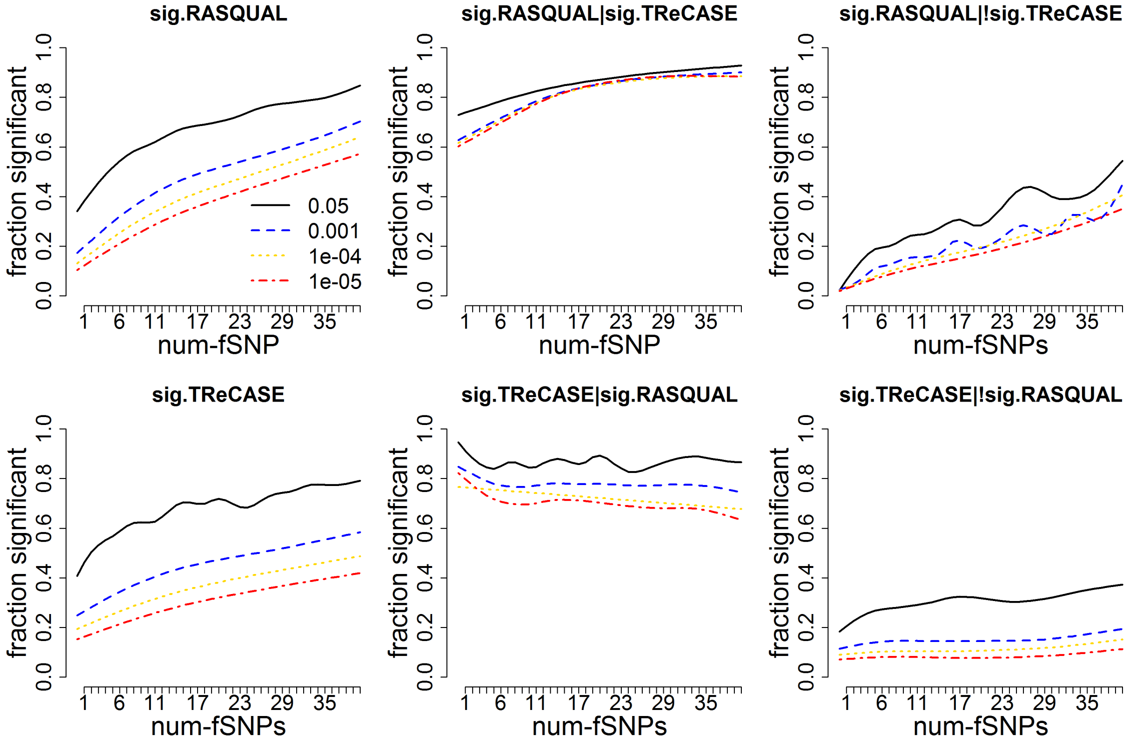

Fig. A31: Method discrepancy conditioned on significance status of each method. We classify the genes into significant or not significant category using FDR cutoffs presented in the legend: 0.05, 1e-3, 1e-4 and 1e-5 plotted vs number of fSNPs. The curve is obtained using a spline. Panels (a) and (d) consider overall dependency of fraction of genes found to be significant plotted versus number of fSNPs. Panel (b) considers proportion of genes passing a cutoff in RASQUAL model for all the genes passing cutoff for TReCASE. Panel (e) does it other way around - fraction of significant genes found by TReCASE among the genes significant in RASQUAL. Panels (c) and (f) provide similar curves for fraction of genes found to be significant by one of the methods, given that they weren't found to be significant by the other method.

C.7.6 eQTL mapping using permuted genotype data

In this subsection, we evaluate potential type I error inflation of TReCASE and RASQUAL using permuted genotype data. We only include the potential *cis*-acting eQTLs in our evaluation because ASE is only informative for *cis*-eQTL mapping. To identify *cis*-acting eQTLs, we test whether the eQTL effects estimated by TReC and ASE are the same by a cis-trans test [9], and consider the cases with cis-trans test p-value > 0.05 as the potential *cis*-acting eQTLs. For TReCASE method, we consider the standard TReCASE using likelihood ratio test (LRT) [9] as well as another version using score test [23].

From the distribution of all the eQTL p-values, it is clear that RASQUAL has severely inflated type I error (Fig A32(c,f)). This is consistent with our simulation results (Section C.6.4) and comparison of RASQUAL and TReCASE using the Geuvadis dataset (Section C.7.5). With sample size of 280, the p-value distribution from TReCASE (score) is slightly deviated from uniform distribution (Fig A32(a,d)), though such deviation becomes larger for smaller sample size of 100 (Fig A33(a,d)). Therefore, when using TReCASE (score) method for small sample size, we recommend using permutation p-values rather than the p-values from asymptotic distribution.

We observed that TReCASE also has slight inflated type I error. This may be due to model mis-specification for some genes, either because the distribution assumption is not accurate or missing some covariates (e.g., genetic effect are missing after permuting genotype data). Such inflation of type I error can be removed after trimming outlier values using an approach implemented in DESeq2 [24]. An observation is defined as an outlier if it Cook's distance is larger than a thresholds, and found the threshold of $4/n$ [25] effectively removes the inflation of type I error (Fig A34).

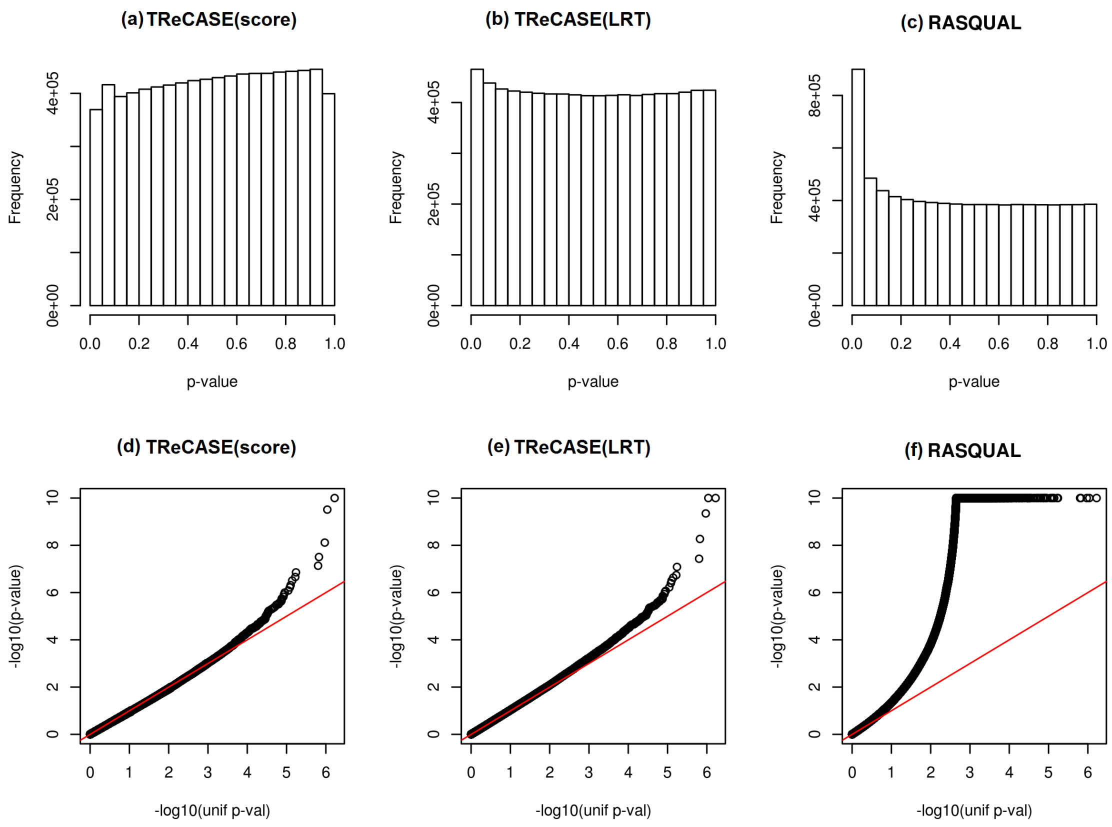

Fig. A32: The distributions ((a)-(c)) and QQ-plots ((d)-(f)) of eQTL p-values using permuted genotypes by three methods: TReCASE (LRT), TReCASE(Score) and RASQUAL, using Geuvadis dataset with sample size 280.

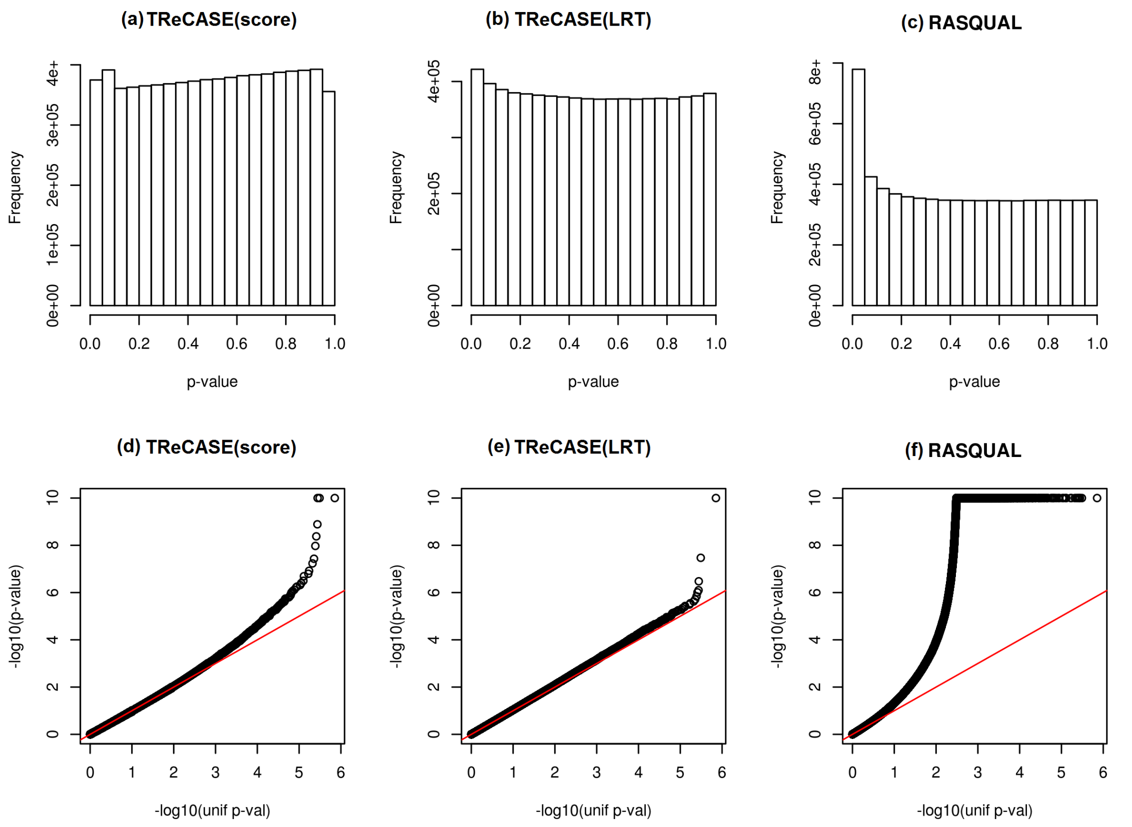

Fig. A33: The distributions ((a)-(c)) and QQ-plots ((d)-(f)) of eQTL p-values using permuted genotypes by three methods: TReCASE (LRT), TReCASE(Score) and RASQUAL, using Geuvadis dataset with sample size 100.

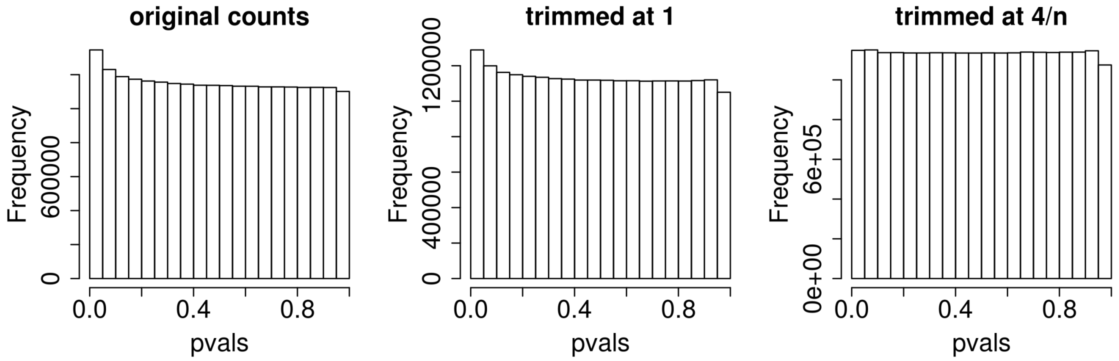

Fig. A34: Fitting the data with permuted genotypes using TReC model after trimming counts with significant Cook's distances

C.8 Compare MatrixEQTL vs. TReCASE using Geuvadis and GTEx v7 data

As mentioned earlier in Section C.7.1, it is computationally infeasible to run RASQUAL on GTEx data. Due to the large number of feature SNPs in GTEx data, RASQUAL can be 100 times slower than TReCASE. Here we make a systematic comparison of TReCASE versus MatrixEQTL using both Geuvadis and GTEx v7 data.

In Geuvadis dataset with permutation p-values estimated by geoP, MatrixEQTL was able to identify 3,336 genes at $q = 0.01$ cutoff versus 6,740 found by TReCASE (Table A15). If the permutation p-values are estimated using eigenMT, MatrixEQTL results identify only 2,773 genes to be significant at q-value 0.01. This is because eigenMT is more conservative than geoP for the permutation p-values that correspond to q-value of 0.01. The estimated proportion of nulls using gene-specific permutation p-values are 23.5%, 41.5%, and 94.2% for TReCASE (geoP) , MatrixEQTL (geoP), and MatrixEQTL (eigenMT), respectively.

Table A15: Number of genes passing corresponding q-value cutoffs (that were estimated using permuted p-values estimated by geoP) in Geuvadis dataset by TReCASE vs MatrixEQTL.

MatrixEQTL<br>TReCASE	$[0.01, 1]$	$[10^{-3}, 0.01)$	$[10^{-4}, 10^{-3})$	$[10^{-5}, 10^{-4})$	$<$ 1e-5	total
$[0.01, 1]$	7543	167	39	16	41	7806
$[10^{-3}, 0.01)$	1727	223	61	26	26	2063
$[10^{-4}, 10^{-5})$	666	147	70	35	55	973
$[10^{-5}, 10^{-4})$	416	104	72	42	88	722
$<$ 1e-5	858	298	241	223	1362	2982
total	11210	939	483	342	1572	14546

GTEx dataset (v7, whole blood) shows more dramatic gain from MatrixEQTL to TReCASE (Table A16) with only 1,878 genes passing $q = 0.01$ cutoff for MatrixEQTL + geoP versus 7,850 found by TReCASE + geoP. Applying eigenMT to MatrixEQTL results would make the results even more conservative declaring only 1,662 genes to be significant at $q = 0.01$. The estimated proportion of nulls using gene-specific permutation p-values are 21.2%, 57.4%, and 100% for TReCASE (geoP), MatrixEQTL (geoP), and MatrixEQTL (eigenMT), respectively.

Table A16: Number of genes passing corresponding q-value cutoffs (that were estimated using permuted p-values estimated by geoP) in GTEx dataset (v7 whole blood) by TReCASE vs MatrixEQTL.

MatrixEQTL	$[0.01, 1]$	$[10^{-3}, 0.01)$	$[10^{-4}, 10^{-3})$	$[10^{-5}, 10^{-4})$	< 1e-5	total
TReCASE						
$[0.01, 1]$	8721	36	11	2	7	8777
$[10^{-3}, 0.01)$	2067	71	26	11	9	2184
$[10^{-4}, 10^{-5})$	927	78	28	15	11	1059
$[10^{-5}, 10^{-4})$	543	58	30	20	28	679
< 1e-5	2491	264	212	152	809	3928
total	14749	507	307	200	864	16627

Next, we study whether the eSNPs (the most significant eSNPs per gene) found by MatrixEQTL and TReCASE are located in different genomic regions, in terms of the 18 chromatin states classification provided by Roadmap Epigenomic Consortium [26]. We consider the results of the two methods are concordant if their eSNPs of the same gene are within 10kb. Considering the SNPs significant at permutation p-value $\alpha = 0.01$ level and contrasting them to the genes non-significant (at $\alpha = 0.1$ level). Then each gene can be assigned to one of 8 categories based on 3 factors, whether the eSNPs found by MatrixEQTL and TReCASE are concordant and whether the eQTL association is significant for each method. We observed the distribution of these 8 groups has large difference for a few chromatin states (Fig A35). The eQTLs identified by both methods or by TReCASE only are less likely located in the Weak Repressed PolyComb (ReprPCwk) or Quiescent/Low (Quies) regions. We observed similar patterns in the results from GTEx (v7 whole blood) dataset (Fig A36).

Alternatively, we considered a different classification from Roadmap Epigenomic Consortium [26] in which certain DNase enriched regions were classified as promoter or enhancer. For these genes we estimated the probability of the SNP falls into a promoter or enhancer region based on the significance level by each method and distance from the gene to the corresponding eSNP (Table A17). We observe that in both datasets stronger TReCASE p-value is associated with higher chance to be

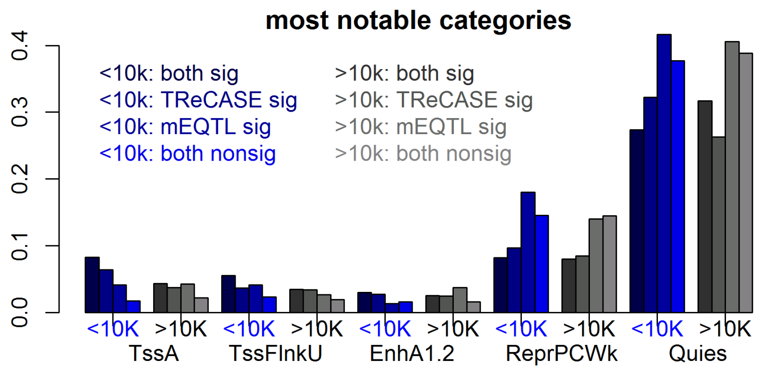

Fig. A35: Distribution of eQTLs (from Geuvadis dataset) in different chromatin states. Here mEQTL stands for MatrixEQTL. For each of the 8 categories labeled in the top-left of the plot, we calculated the proportion of eQTLs located in each of the 18 chromatin states. Only the 5 chromatin states with larger difference across the 8 categories are shown. These groups are: (1) TssA - Active TSS, (2) TssFlnkU - Flanking TSS Upstream, (3) EnhA1 - Active Enhancer 1 and Active Enhancer 2, (4) ReprPCwk - Weak Repressed PolyComb and (5) Quies - Quiescent/Low

located within enhancer or particularly promoter while the signal for MatrixEQTL is weaker to non-existent. This observation suggests that incorporation of ASReC not only increases power, but also improves precision.

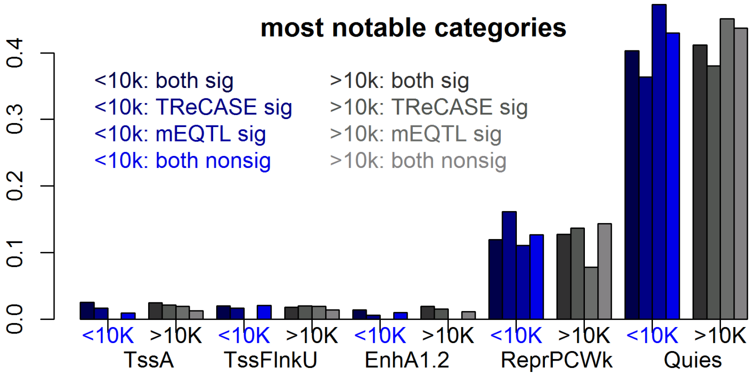

Fig. A36: Distribution of eQTLs (from GTEx v7 whole blood dataset) in different chromatin states. The categories are the same as Fig A35.

Table A17: Association between eQTL signals and promoter or enhancer status. We fit a logistic regression with promoter/enhance status as response variable and with eQTL p-values and distance between the gene and the corresponding eSNP as predictors. The eQTL p-values are on negative $\log_{10}$ scale, and distance is on $\log_{10}$ scale with 1 added. The distance from a gene to a SNP within this gene is considered to be 0.

	Geuvadis				GTEx v7 whole blood			
	promoter		enhancer		promoter		enhancer	
	coef	p-val	coef	p-val	coef	p-val	coef	p-val
Intercept	-2.5	0	-3.21	8e-290	-3.7	4e-254	-3.6	8e-253
p_{mEQTL}	0.01	0.37	-0.02	0.07	-5e-5	0.99	-0.02	0.07
$p_{TReCASE}$	0.05	8e-5	0.02	0.001	0.011	0.03	0.01	0.0003
distance	-0.06	1e-15	-0.005	0.58	-0.014	0.18	0.003	0.76

References

1. 1000 Genomes Project Consortium. A global reference for human genetic variation. Nature. 2015;526(7571):68.

2. Lappalainen T, Sammeth M, Friedländer MR, Ac‘t Hoen P, Monlong J, Rivas MA, et al. Transcriptome and genome sequencing uncovers functional variation in humans. Nature. 2013;501(7468):506–511.

3. Delaneau O, Marchini J, Consortium GP, et al. Integrating sequence and array data to create an improved 1000 Genomes Project haplotype reference panel. Nature communications. 2014;5.

4. Howie B, Marchini J, Stephens M. Genotype imputation with thousands of genomes. G3: Genes, Genomes, Genetics. 2011;1(6):457–470.

5. Howie BN, Donnelly P, Marchini J. A flexible and accurate genotype imputation method for the next generation of genome-wide association studies. PLoS Genet. 2009;5(6):e1000529.

6. Rhead B, Karolchik D, Kuhn RM, Hinrichs AS, Zweig AS, Fujita PA, et al. The UCSC genome browser database: update 2010. Nucleic acids research. 2009; p. gkp939.

7. Purcell S, Neale B, Todd-Brown K, Thomas L, Ferreira MA, Bender D, et al. PLINK: a tool set for whole-genome association and population-based linkage analyses. The American Journal of Human Genetics. 2007;81(3):559–575.

8. Trapnell C, Pachter L, Salzberg SL. TopHat: discovering splice junctions with RNA-Seq. Bioinformatics. 2009;25(9):1105–1111.

9. Sun W. A statistical framework for eQTL mapping using RNA-seq data. Biometrics. 2012;68(1):1–11.

10. Lawrence M, Huber W, Pages H, Aboyoun P, Carlson M, Gentleman R, et al. Software for computing and annotating genomic ranges. PLoS Comput Biol. 2013;9(8):e1003118.

11. McKenna A, Hanna M, Banks E, Sivachenko A, Cibulskis K, Kernytsky A, et al. The Genome Analysis Toolkit: a MapReduce framework for analyzing next-generation DNA sequencing data. Genome research. 2010;20(9):1297–1303.

12. GTEx Consortium. Genetic effects on gene expression across human tissues. Nature. 2017;550(7675):204–213.

13. Consortium G, et al. The GTEx Consortium atlas of genetic regulatory effects across human tissues. Science. 2020;369(6509):1318–1330.

14. Frankish A, Diekhans M, Ferreira AM, Johnson R, Jungreis I, Loveland J, et al. GENCODE reference annotation for the human and mouse genomes. Nucleic acids research. 2019;47(D1):D766–D773.

15. Paul SR, Balasooriya U, Banerjee T. Fisher Information Matrix of the Dirichlet-multinomial Distribution. Biometrical Journal: Journal of Mathematical Methods in Biosciences. 2005;47(2):230–236.

16. Kumasaka N, Knights AJ, Gaffney DJ. Fine-mapping cellular QTLs with RASQUAL and ATAC-seq. Nature genetics. 2016;48(2):206–213.

17. Ongen H, Buil A, Brown AA, Dermitzakis ET, Delaneau O. Fast and efficient QTL mapper for thousands of molecular phenotypes. Bioinformatics. 2016;32(10):1479–1485.

18. Newman AM, Liu CL, Green MR, Gentles AJ, Feng W, Xu Y, et al. Robust enumeration of cell subsets from tissue expression profiles. Nature methods. 2015;12(5):453–457.

19. Newman AM, Steen CB, Liu CL, Gentles AJ, Chaudhuri AA, Scherer F, et al. Determining cell type abundance and expression from bulk tissues with digital cytometry. Nature biotechnology. 2019;37(7):773–782.

20. Zhabotynsky V, Inoue K, Magnuson T, Calabrese JM, Sun W. A Statistical Method for Joint Estimation of Cis-eQTLs and Parent-of-Origin Effects under Family Trio Design. Biometrics. 2019;.

21. Tarone RE. Testing the goodness of fit of the binomial distribution. Biometrika. 1979;66(3):585–590.

22. Shabalin AA. Matrix eQTL: ultra fast eQTL analysis via large matrix operations. Bioinformatics. 2012;28(10):1353–1358.

23. Hu YJ, Sun W, Tzeng JY, Perou CM. Proper Use of Allele-Specific Expression Improves Statistical Power for cis-eQTL Mapping with RNA-Seq Data. Journal of the American Statistical Association. 2015;110(511):962–974.

24. Love MI, Huber W, Anders S. Moderated estimation of fold change and dispersion for RNA-seq data with DESeq2. Genome biology. 2014;15(12):550.

25. Hardin JW, Hardin JW, Hilbe JM, Hilbe J. Generalized Linear Models and Extensions, Second Edition. A Stata Press publication. Taylor & Francis; 2007. Available from: https://books.google.com/books?id=tOeqO6Hs-6gC.

26. Kundaje A, Meuleman W, Ernst J, Bilenky M, Yen A, Heravi-Moussavi A, et al. Integrative analysis of 111 reference human epigenomes. Nature. 2015;518(7539):317.
