# Supplementary material for: eQTL mapping using allele-specific count data is computationally feasible, powerful, and provides individual-specific estimates of genetic effects
Source: PLoS Genet. 2022 Mar 14;18(3):e1010076. doi: 10.1371/journal.pgen.1010076 (PMC8947591; doi:10.1371/journal.pgen.1010076)
Supplement: Attachment — Submitted filename: response.docx [file pgen.1010076.s008.docx]

**Comments to the Authors:**

**Reviewer #1:**

Zhabotynsky et al. present geoP, a new method to approximate permutation p values, to reduce the computational burden of permutation tests in detecting eQTLs with allele-specific gene expression (ASE). The geoP method approximates the permutation p values by estimating the relationship between permutation p values and minimum nominal p values per gene. Overall, the manuscript is well written, and this work has implications for enabling eQTL detection with ASE in large-scale eQTL studies. The authors have devoted efforts in present analyses on 28 GTEx tissues and Geuvadis data. The authors also developed a data processing pipeline to process the RNAseq data saved on cloud. Clarifications and changes that could improve the paper include:

*Thank you for reviewing our paper. We have highlighted the changes in the “Revised Article with Changes Highlighted”, which should be at the end of the combined pdf document.*

Major:
1. The authors compared the number of eGenes identified by different methods in the real datasets. As the same eGene could be affected by more than one functional regulatory variant, I was wondering for the same gene-SNP pairs tested, how many significant pairs are consistent between different methods, and how many pairs are additionally identified by TReCASE equipped with geoP? How would the results change with different permutation p-value cutoffs?

*Thank you for your suggestion. In the revised manuscript, we have compared the number of gene-SNP pairs identified by TReCASE and MatrixEQTL. We have reported our results at Supplementary Materials Section C.3.1, and added the following paragraph in the revised manuscript (page 8):*

*“We also compared the number of gene-SNP pairs identified by MatrixEQTL and TReCASE and their intersections. At permutation p-value cutoff 0.01, the vast majority (96%-98%) of the gene-SNP pairs identified by MatrixEQTL can be identified by TReCASE, and number of additional gene-SNP pairs identified by TReCASE ranges from 38% to 100% of the number of gene-SNP pairs identified by MatrixEQTL (Supplementary Figures 8-9). The results are similar across several permutation cutoffs (Supplementary Figure 10).”*

2. The authors showed the functional categories of the top eQTLs identified by TReCASE and MatrixEQTL are consistent with each other. It would be more convincing to provide additional biological insights by TReCASE beyond the consistent findings.

*Yes, we agree. We should have clarified that the reason we compare the functional category enrichment is to show those additional TReCASE eQTLs are as “genuine” as the eQTLs identified by MatrixEQTL. We have added such clarification in the revised manuscript (page 8). In addition, we have conducted a comprehensive assessment on whether GWAS hits are enriched among eQTLs. We not only considered all the GWAS hits but also divided them into 21 categories. We did find in some cases, the GWAS hit of some categories were enriched among the TReCASE eQTLs but not the MatrixEQTL eQTLs. We have added summary of our results in main text (page 9) as well as Section C.3.2. of the Supplementary Materials.*

3. In dynamic eQTL detection, what is the biological interpretation for the identified dynamic eQTLs? For example, how would the effect size of eQTLs change over different expression levels of CTCF or TP53? What functional categories are these dynamic eQTLs enriched in? Are these dynamic eQTLs also detected as eQTLs in the original GTEx study or TReCASE?

*Thank you for these great questions. Sorry that our presentation in the previous version of our manuscript was clear. We indeed only studied dynamic eQTLs for the eGenes with permutation p-value smaller than 0.01 and focus on the SNP with smallest p-value for each eGene. We mentioned this in the Method section but failed to clarify it in the main text. We have added the following text in the revised manuscript (2^nd^ paragraph, page 11):*

*An in-depth study of dynamic eQTLs warrant separate works tailored to the contents of interest. Here we mainly want to use some simple examples to illustrate that ASE has the power to deliver individual-specific eQTL effect estimates, which are very useful source to study dynamic eQTLs. We reason that when there are dynamic eQTLs, we should also see eQTL signals without conditioning on relevant content. In fact, this is not a stringent requirement given that around 50-70\% of all genes tested are identified as eGenes by TReCASE across the 28 GTEx tissues in our study. For each eGene, we only studied the dynamic eQTL potential for the SNP with strongest marginal eQTL signal.*

*Since the number of dynamic eQTLs identified are very few, it is hard to assess functional enrichment. We did evaluate whether the CTCF dynamic eQTLs are enriched with CTCF binding sites (CTCFBSDB 2.0 http://insulatordb.uthsc.edu) or whether the TP53 dynamic eGenes are enriched with TP53 targets. No significant enrichment was found in either case. We have reported such findings at the end of Dynamic eQTL section of the revised manuscript (page 12).*

4. A recently published paper (Liang et al, 2021, NC) developed a method named mixQTL that can also identify eQTLs with allele-specific expression and is scalable to large sample sizes. What are the benefits of TReCASE equipped with geoP over mixQTL?

*Thank you for pointing out this very relevant recent work. MixQTL does use allele-specific expression to improve the power of eQTL mapping. To improve computational efficiency, it uses a linear model framework and assumes the log ratio of allele-specific counts from the two haplotypes follows a normal distribution. This assumption is likely not accurate when the counts are relatively low. Therefore, the power improvement by mixQTL versus methods that do not use ASE (e.g., MatrixEQTL) is likely more apparent for genes with higher expression. In fact, when they compared mixQTL vs. methods not using allele-specific expression using GTEx whole blood data (in the section “mixQTL outperforms standard eQTL mapping in GTEx data”), they considered* ***5,734 genes*** *for which (1) at least 15 samples having at least 50 allele-specific counts for each haplotype; and (2) at least 500 samples having a total read count of at least 100. In contrast, TReCASE can handle genes with much smaller number of allele-specific counts. We used* ***16,290 genes*** *with at least 5 samples having at least 5 allele-specific counts. Therefore, count based models like TReCASE can have higher power than mixQTL since they can more effectively exploit ASE in more genes. We have added the discussion in the revised manuscript, in the last paragraph of the Discussion section (page 16).*

*The mixQTL work did not use permutation p-value. It would be very valuable to use permutation p-value because local SNPs of a gene tend to have strong LD and permutation p-value can account for such dependence. Our geoP method estimates the effective number of independent tests and it is independent with the TReCASE method. Therefore, the geoP method can be used to calculate permutation p-values for mixQTL.*

5. It would be easier for other people to use your method if it is implemented in software packages. Also, the current description of this method on page 16 is not clear enough. For example, the authors mentioned “do additional adjustment and restart the process”, what kind of “additional adjustment” is suggested?

*Yes, it is indeed implemented in an R package asSeq. The permutation p-value estimation is implemented in function “getPermP”. Sorry for the very vague description “do additional adjustment and restart the process”. We have modified that part (page 18) to clearly describe what are the additional adjustments.*

Minor:
1. In Figure 3e-f, the authors compared functional category enrichment of eQTLs in enhancers in 5 tissues but compared all 28 tissues in figure 3a-d. Why only concentrate on these 5 tissues in figure 3e-f?

*This is because the tissue-specific enhancer data are available only for these 5 tissues from the EnhancerAtlas 2.0 database. We have clarified this point at page 9.*

2. In figure 5f, why is the detection power increased with decreased genetic effect? Figure 5f is not mentioned in the main text.

*Thank you for catching this mistake. The x-axis labels were wrong, and we have fixed it. Now the power increases with genetic effect. We have also added citation to Figure 5f (page 15, before the Discussion section).*

**Reviewer #2:**

The authors proposed a geoP method which they claimed to be computationally more efficient and robust at various cutoffs than the eigenMT does in eQTL mapping using ASE information. They also demonstrated the usage of ASE to study dynamic eQTLs and compared two popular methods TReCASE versus RASQUAL. Though the authors did a lot of analyses with various GTEx data sets, I feel the organization and presentation need to be further improved to demonstrate the major contributions of the work. What’s the major contribution and the novelty of the work? They need to be clearly presented and illustrated. I understand the authors try to demonstrate the use of ASE in eQTL mapping. However, the two topics covered in the work, developing a geoP method and studying dynamic eQTLs, do not seem providing a strong support of the title of the work (in fact, they seem to be unrelated). The title is also too broad and not specific.

*Thank you for reviewing our paper. We have highlighted the changes in the “Revised Article with Changes Highlighted”, which should be at the end of the combined pdf document.*

*Thank you for these great suggestions. The main contribution of our work is to make eQTL mapping with ASE computationally feasible for large datasets (by the introduction of geoP method) and demonstrate the benefit to include ASE in eQTL mapping: improved power and an additional advantage to obtain individual-specific estimates of genetic effects, which can be used to study dynamic eQTLs. We have modified our title to be: “eQTL mapping using allele-specific count data is computationally feasible, powerful, and provides individual-specific estimates of genetic effects”.*

The abstract needs to be polished to summary the major contribution of the work.

*Thank you. We have rewritten the abstract to summary our major contributions.*

It is not convincible by the way the authors defined false negatives and false positives shown in Figure 1(c). This is also related to what the authors claimed: “Using a permutation p-value cutoff of 0.01 (corresponding to FDR around 1%), we detected 20-100% more eGenes (genes with at least one significant eQTL) than the most recent GTEx study [1], where ASE was not used in eQTL mapping.” Is this claim based on real data analysis or simulation? If based on real data analysis, then it is not convincible. A method that detects more eQTLs than others does not imply that it is more powerful based on the real data analysis. How do you know what you detected are not false positives? geoP does look like have higher false positives than eigenMT does from Fig 1(c) (again, not sure if the figure is based on simulation or real data analysis). To claim a method is more powerful than others, one should do a simulation study in which the underlying truth is known.

*We agree that identifying more eGenes in real data should not be interpreted as improved power since it is possible the detected eGenes are false positives. We have modified our text to avoid using words “power gain” and just state the fact that more eGenes are found. We have also used simulation studies to evaluate the power gain. We have added the following paragraph in the revised manuscript (starting from the end of page 7):*

*” Additional findings from real data may not indicate power gain, but due to larger number of false discoveries. We have conducted simulation studies with different effect sizes and sample sizes to demonstrate for typical eQTL effect sizes observed in GTEx data, TReCASE can indeed reach more than 100% power gain than MatrixEQTL (Section C.2. of Supplementary Materials).”*

*More details of simulation setup and results are presented in Section C.2 of the revised Supplementary Materials.*

The authors stated in the Discussion “Although similar message has been shown in earlier studies, our results are more comprehensive due to the larger number of datasets that we have studied.” Analyzing more datasets does not add to the novelty of the work.

*We agree that analyzing more datasets does not add the novelty of the work. We have removed this sentence.*

The authors spent large efforts in building an online cloud-based data processing pipeline. This is much appreciated.

*Thank you!*

**Reviewer #3:** Review of Zhabotynsky et al.

In this study, Zhabotynsky et al describe and test a method for eQTL mapping that leverages allele-specific read counts to increase mapping power. This is not a novel approach, but the authors do a good job showing that their method is faster and more accurate than other approaches. Overall, I found this paper to be very clearly written with well-justified analyses. This is a very impressive demonstration of the power of utilizing ASE and well-designed statistical modeling to identify eQTL.

*Thank you for reviewing our paper. We have highlighted the changes in the “Revised Article with Changes Highlighted”, which should be at the end of the combined pdf document.*

Detailed Comments:

1. The authors compare their methods to those reported in the GTEx paper. They note some differences in processing and testing pipeline and write “Despite these minor differences, the number of eGenes reported by the two pipelines (when we use MatrixEQTL for eQTL mapping) are highly consistent (Figure 2a).”. I don’t think Figure 2a shows this. I think Figure 2a needs to have GTEx on one axis and MatrixEQTL on the other in order to support the point they are making. Or is this an axis labelling error and the figure does show what they say? In addition to this figure it would be good to report (numerically) the percentage overlap in eQTL reported by the GTEx paper compared to those discovered using these authors’ pipeline with MatrixEQTL. I assume the overlap is >90-95%.

*Sorry, it was a wrong axis label in Figure 2a. The x-axis for Figure 2(a) is indeed the number of eGenes by GTEx. We have also reported the percentage overlap between the eGenes reported by GTEx and our MatrixEQTL pipeline. The total number of eGenes identified by MatrixEQTL ranges 90% to 100% (median 98%) of the number of eGenes identified by GTEx. The percentage of overlaps among all the GTEx eGenes ranges from 76% to 90%, with median of 86%. The overlap is not extremely high because we consider different genomic regions to search for eQTLs and this choice also affect the calculation of permutation p-values. For example, long genes have larger number of local SNPs and larger number of independent tests. We have added these numerical summary and explanation in the revised manuscript (page 6).*

2. Figure 2a-c what are dotted red lines? It looks like the line in (a) is a best fit regression line, and that this same line is plotted in (b)? The line in (c) looks different but I think that is just due to different axis limits. I suggest harmonizing axis limits on both axes for subfigures (a) through (c)

*Sorry that we did not explain the dotted red lines. We have modified the figure captions to add explanations. They are the same across all three panels and they indicate a reference line of y=x. We tried to harmonize the axis limits though it would leave a considerate amount of white space in panel (a). Since the refence line y=x provides additional information to align the three panels, we did not change the axis limits.*

3. In the section “Explore dynamic eQTLs using individual-specific genetic effects estimated by ASE” (note: typo in section title, should be “Exploring”), I’m not sure I understand the point of including the models that condition on CTCF and TP53. The authors report some interesting results on age-associated dynamic eQTL in blood, but don’t report anything of note regarding the CTCF and TP53 models in the main text.

*Thank you for these great questions and pointing out this typo, which we have fixed in the revision. Yes, we did not give a good explanation on why including CTCF and TP53 in the previous version of our manuscript. Our motivation is to show that eQTL mapping with ASE not only improves power, but also delivers additional information of individual-specific estimates of genetic effects. We meant to only illustrate such analysis from a couple examples. We have added the following clarification at page 11:*

*“An in-depth study of dynamic eQTLs warrants separate works tailored to the contents of interest. Here we mainly want to use some simple examples to illustrate that ASE has the power to deliver individual-specific eQTL effect estimates, which are very useful source to study dynamic eQTLs.”*

*We want to include some transcriptional factors because some eQTLs may be transcriptional factor binding sites. The inclusion of CTCF and TP53 are somewhat arbitrary. It is simply because they are two well studied transcription factors. We sought to check whether the CTCF dynamic eQTLs are enriched with CTCF binding sites or whether the TP53 dynamic eGenes are enriched with TP53 targets, though we did not observe significant enrichment in either case, which could be partly due to relatively small number of dynamic eQTLs. We have reported such findings at the end of Dynamic eQTL section of the revised manuscript (page 12).*

4. I think it would be very useful if the authors could include some more detailed information on eQTL that they are able to detect that were missing from the MatrixEQTL analysis. For example, they could present a detailed portrait of a single eGene where they show the actual data and illustrate why the ASE and beta-binomial modeling are pulling this eGene to significance.

*Thank you for your suggestion. We have included such examples in Supplementary Figures 11-14, in Section C.3.2 of Supplementary Materials. We have mentioned such examples in the revised main text (the end of 2^nd^ paragraph on page 8).*

5. To improve accessibility/usability, I suggest that the authors upload their singularity image to a hub/database such as [https://cloud.sylabs.io/](https://urldefense.proofpoint.com/v2/url?u=https-3A__cloud.sylabs.io_&d=DwQGaQ&c=eRAMFD45gAfqt84VtBcfhQ&r=4M-R7nl8FR0HQIhWzmBoykKdZt1BDwxm084eD6Ntm6E&m=amITiz0yyiCZf74-7Wk5VY_Td3nFXjaZPofLHN1nrag&s=p2TFzss-5wlqP5--g07dSEutxcNQoucab4qA8dUtoJE&e=). Although there pipeline appears to be available at [https://github.com/Sun-lab/gtex_AnVIL](https://urldefense.proofpoint.com/v2/url?u=https-3A__github.com_Sun-2Dlab_gtex-5FAnVIL&d=DwQGaQ&c=eRAMFD45gAfqt84VtBcfhQ&r=4M-R7nl8FR0HQIhWzmBoykKdZt1BDwxm084eD6Ntm6E&m=amITiz0yyiCZf74-7Wk5VY_Td3nFXjaZPofLHN1nrag&s=gRv6vQnygYmlGMSbpU1k-Chg525MJf3zogwyZhFUhF8&e=), there are instructions for other aspects of analysis included (e.g. cloud analysis) that some users may not use. It would be useful to develop a short example walkthrough for the software.

*Thank you for your suggestion. We have studied singularity versus Docker as solution of containerization. Singularity has advantage to be used in computing clusters due to security/user permission concerns. However, singularity only has a beta version for Mac, which is our main platform for local testing, and our cloud environment at AnVIL only supports Docker. Therefore, we prefer to use Docker as containerization for now and we are open to expand it to singularity later. We have built a docker image “sunway1999/bioconductor_trecase:0.1” which is available at DockerHub. We have also packaged all our R script in one file, so that a user can run our pipeline in a local computing environment or a computing cluster by a one-line command. We have described our docker image, workflow for cloud environment and command line for a local/cluster computing environment in the README file of* [*https://github.com/Sun-lab/gtex_AnVIL*](https://github.com/Sun-lab/gtex_AnVIL)*.*
