# Supplementary material for: eQTL mapping using allele-specific count data is computationally feasible, powerful, and provides individual-specific estimates of genetic effects
Source: PLoS Genet. 2022 Mar 14;18(3):e1010076. doi: 10.1371/journal.pgen.1010076 (PMC8947591; doi:10.1371/journal.pgen.1010076)
Supplement: Attachment — Submitted filename: response_R2.docx [file pgen.1010076.s009.docx]

**Comments to the Authors:**

**Reviewer #1:**

I am satisfied with most of the review comments that the authors have addressed, except the results of CTCF and TP53 related dynamic eQTLs. In the updated manuscript, the authors "assessed whether the dynamic eQTLs related with CTCF are enriched among CTCF binding sites (CTCFBSDB 2.0 http://insulatordb.uthsc.edu/ [23]) and whether p53’s target genes (Supplementary Table 3 of [24]) are enriched among the eGenes with dynamic eQTLs with respect to TP53", however, "No significant enrichment was found in either case". The authors chose to study CTCF and TP53 based on the assumption that "TF expression can modulate the strength of eQTLs located in TF binding sites", but the current results do not support this assumption. For the CTCF dynamic eQTLs located in the CTCF binding sites, are their effect sizes more correlated with CTCF expression than other eQTLs? If not, can you find more evidence to support that your identified dynamic eQTLs are truly meaningful?

*Thank you for your comments and suggestions. We totally agree that we need to provide more satisfactory explanation for these results. We have performed additional analysis and examination.*

*First, we suspected that the reason we did not find significant enrichment is because both CTCF and TP53 regulate the expression of many genes, directly or indirectly, which makes it very hard to obtain an accurate list of target genes or binding sites for a particular tissue or condition. For the CTF binding sites, we summarize the size of binding sites annotation from CTCFBSDB 2.0 http://insulatordb.uthsc.edu/ [23], see the following figure.*

*Although most of them is around a few hundred based pairs, some binding sites are extremely wide. In total, these annotated binding sites cover more than 9 million base pairs and 28.9% of the whole genome. This may explain why we cannot find good enrichment. Even if we only consider 200 base pair regions around the center of each annotated binding sites, they cover more than 2 million base pairs and around 7% of the whole genome. This is probably because CTCF acts as an insulator that defines the boundaries between active and heterochromatic DNA and its function is highly un-specific.*

*For the TP53, the target genes from “Supplementary Table 3 of [24]” is a short list of high confidence and direct targets of TP53. TP53, as a master regulator, can regulate many more genes indirectly.*

*Next, following your suggestion, we check “For the CTCF dynamic eQTLs located in the CTCF binding sites, are their effect sizes more correlated with CTCF expression than other eQTLs?”. The conclusion is that there is no significant difference. Again, we think this is because the annotation of CTCF binding sites is highly un-specific.*

*Finally, we seek to use alternative annotations to we examined the transcription factor target genes from a popular database: JASPAR (*[*http://jaspar.genereg.net/*](http://jaspar.genereg.net/)*). The annotation data, which was harmonized by harmonizome (https://maayanlab.cloud/Harmonizome/resource/Jaspar+PWMs) is a big matrix of size 21548x114, for 21548 target genes and 114 transcription factors. There are 2,849 targets for TP53 and only 35 targets for CTCF. Using this annotation, we did find significant enrichment of TP53 targets among TP53-associated dynamic eGenes. Among 130 genes whose eQTL strength were associated with TP53 expression, 22 were TP53 targets while 14 were expected by chance (p-value 0.0497). Since the number of targets for CTCF was very small, we did not find any significant enrichment.*

*We have revised our paper to report these findings and interpretations, at the last paragraph of page 12. Here is the paragraph for your convenience:*

*We have also assessed whether the genes with dynamic eQTLs with respect to CTCF and TP53 are more likely to be their target genes, as defined by JASPAR database [23]. The annotation data, which was harmonized by harmonizome [24], is a big matrix of size 21,548×114, for 21,548 target genes and 114 transcription factors. There are 2,849 targets for TP53 and only 35 targets for CTCF. Using this annotation, we found significant enrichment of TP53 targets among our dynamic eGenes. Among the 130 genes whose eQTL strength were associated with TP53 expression, 22 were TP53 targets while 14 were expected by chance (p-value of Chi-squared test 0.0497). Since the number of targets for CTCF was very small, no significant enrichment was found. We also explored the annotated CTCF binding sites (CTCFBSDB 2.0 http://insulatordb.uthsc.edu/ [25] and evaluated the overlap between CTCF-associated dynamic eGenes and CTCF binding sites. We did not find any significant overlap. We suspect this is because the CTCF binding sites are highly unspecific. They cover around 28.9% of the whole genome. Even if we only consider a region of 200 base pair around the center of each annotated binding site, they cover around 7% of the whole genome and the overlap remains insignificant. These results highlight the challenges to interpret the dynamic eQTL results and we expect that additional data and annotation, such as tissue specific activity of transcription factor protein activities (instead of their gene expression) and tissue-specific annotation of target genes, can improve the accuracy and interpretability of the dynamic eQTL results.*

**Reviewer #2:**

Reviewer #2: The authors have addressed my comments and I have not further comment.

**Reviewer #3:**

The authors have largely addressed my concerns.

I have two minor suggestions:

(1) I like that they show in Supplemental section C.3 the different examples of eQTL that are missed by MatrixEQTL but captured by TReCASE. But please clarify what part (b) is showing in Supp Figures 11, 12, 13, and 14.

*Sorry, we did not clarify panel (b) in the previous revision. We have added the following description in the revised manuscript:*

*Panel (b) illustrates the percentage of allele-specific counts coming from the haplotype associated with the B allele of the eQTL. For example, for haplotype configuration 1 (A-h1 and B-h2), it is the proportion of counts from haplotype h2, and for haplotype configuration 2 (A-h2 and B-h1), it is the proportion of counts from haplotype h1. The size of the circles in panel (b) indicates the number of allele-specific reads.*

(2) Pipeline usability. The availability of a docker image and the authors’ newer in-depth walkthrough/README are very useful. I would request one small addition. The README at [https://github.com/Sun-lab/gtex_AnVIL/blob/master/README.md](https://urldefense.proofpoint.com/v2/url?u=https-3A__github.com_Sun-2Dlab_gtex-5FAnVIL_blob_master_README.md&d=DwQGaQ&c=eRAMFD45gAfqt84VtBcfhfEazhEXT91ASHynm_9f1N0&r=4M-R7nl8FR0HQIhWzmBoykKdZt1BDwxm084eD6Ntm6E&m=RtgbNqxc_fgJR4LgCKaoxSMvWoEXXgRW03QplePRQI6DdzFsL3gHcnxm5Y1VFoHw&s=hlTx6EQ2eXlIzmtQwo9cBIetpWks3dBAO74vdQ-pULA&e=) provides information on the four arguments to their script get_TReC_ASReC.R. It would be really useful to have “toy” versions of those arguments that are files added directly to the GitHub repo. Sometimes when one is running a new pipeline it is very useful to test it on small example files just to ensure that the pipeline is working as expected*.*

*Thank you for your suggestion. We totally agree. It is a bit complicated since we need to share raw bam files as well as genotype data. To manage the data size and satisfy data access protection, we included sample data of two genes in 25 samples from 1000 Genome Project in our asSeq_pipelines repository:*

[*https://github.com/Sun-lab/asSeq_pipelines/tree/main/pipeline_1KGP/data*](https://github.com/Sun-lab/asSeq_pipelines/tree/main/pipeline_1KGP/data)

*We have also added a sample R code test_scr.R in the pipeline_1KGP folder of asSeq_pipelines repository to iteratively call function get_TReC_ASReC.R to calculate counts from those bam files.*
